# Evidence for bidirectional and *trans*-synaptic parasympathetic and sympathetic propagation of alpha-synuclein in rats

**DOI:** 10.1007/s00401-019-02040-w

**Published:** 2019-06-26

**Authors:** Nathalie Van Den Berge, Nelson Ferreira, Hjalte Gram, Trine Werenberg Mikkelsen, Aage Kristian Olsen Alstrup, Nicolas Casadei, Pai Tsung-Pin, Olaf Riess, Jens Randel Nyengaard, Gültekin Tamgüney, Poul Henning Jensen, Per Borghammer

**Affiliations:** 1 Institute for Clinical Medicine, Aarhus University, Aarhus, Denmark; 2 Nuclear Medicine and PET, Aarhus University Hospital, Aarhus, Denmark; 3 DANDRITE-Danish Research Institute of Translational Neuroscience and Department of Biomedicine, Aarhus University, Aarhus, Denmark; 4 Institute of Medical Genetics and Applied Genomics, University of Tuebingen, Tuebingen, Germany; 5 IMBA-Institute of Molecular Biotechnology, Vienna, Austria; 6 Center for Stochastic Geometry and Advanced Bioimaging, Aarhus University, Aarhus, Denmark; 7 Institute of Physical Biology, Heinrich-Heine-University, Düsseldorf, Germany; 8 Institute of Complex Systems, Structural Biochemistry (ICS-6), Forschungszentrum Jülich, Jülich, Germany

**Keywords:** Alpha-synuclein, BAC rat model, Prion-like spread, Autonomic nervous system, Parkinson’s disease

## Abstract

The conversion of endogenous alpha-synuclein (asyn) to pathological asyn-enriched aggregates is a hallmark of Parkinson’s disease (PD). These inclusions can be detected in the central and enteric nervous system (ENS). Moreover, gastrointestinal symptoms can appear up to 20 years before the diagnosis of PD. The dual-hit hypothesis posits that pathological asyn aggregation starts in the ENS, and retrogradely spreads to the brain. In this study, we tested this hypothesis by directly injecting preformed asyn fibrils into the duodenum wall of wild-type rats and transgenic rats with excess levels of human asyn. We provide a meticulous characterization of the bacterial artificial chromosome (BAC) transgenic rat model with respect to initial propagation of pathological asyn along the parasympathetic and sympathetic pathways to the brainstem, by performing immunohistochemistry at early time points post-injection. Induced pathology was observed in all key structures along the sympathetic and parasympathetic pathways (ENS, autonomic ganglia, intermediolateral nucleus of the spinal cord (IML), heart, dorsal motor nucleus of the vagus, and locus coeruleus (LC)) and persisted for at least 4 months post-injection. In contrast, asyn propagation was not detected in wild-type rats, nor in vehicle-injected BAC rats. The presence of pathology in the IML, LC, and heart indicate *trans*-synaptic spread of the pathology. Additionally, the observed asyn inclusions in the stomach and heart may indicate secondary anterograde propagation after initial retrograde spreading. In summary, *trans*-synaptic propagation of asyn in the BAC rat model is fully compatible with the “body-first hypothesis” of PD etiopathogenesis. To our knowledge, this is the first animal model evidence of asyn propagation to the heart, and the first indication of bidirectional asyn propagation via the vagus nerve, i.e., duodenum-to-brainstem-to-stomach. The BAC rat model could be very valuable for detailed mechanistic studies of the dual-hit hypothesis, and for studies of disease modifying therapies targeting early pathology in the gastrointestinal tract.

**Electronic supplementary material:**

The online version of this article (10.1007/s00401-019-02040-w) contains supplementary material, which is available to authorized users.

## Introduction

Parkinson’s disease (PD) is the second most common neurodegenerative disease after Alzheimer’s disease. The disorder is characterized by loss of dopaminergic neurons in the substantia nigra (SN) and the presence of Lewy bodies (LBs) and Lewy neurites (LNs) in the central nervous system (CNS) and peripheral nervous system (PNS). Lewy pathology consists of alpha-synuclein (asyn)-enriched aggregates, which are hypothesized to self-amplify and propagate in a prion-like manner through interconnected neurons by the recruitment of soluble endogenous asyn protein [14, 49].

The etiology of the initial asyn aggregates is still largely unknown. The dual-hit hypothesis proposes that the first inclusions form in the olfactory bulb and peripheral autonomic nerve endings of the gut, thereby explaining why the dorsal motor nucleus of the vagus (DMV) seems to be affected early in most cases of PD [9, 10]. This hypothesis is supported by several lines of evidence. Asyn pathology can be detected in the gut of PD patients up to 20 years prior to diagnosis [18, 45, 48], and full truncal vagotomy seems to lower the risk of PD by 40–50% after 10–20 years of follow-up [23, 51]. Also, recent in vivo imaging data showed that prodromal PD patients display marked damage to the parasympathetic and sympathetic nervous system, while their nigrostriatal dopamine system is still relatively intact [22]. Moreover, recent studies support a bidirectional link between gastrointestinal inflammation and the initiation and progression of PD [39]. Nevertheless, the dual-hit hypothesis remains controversial, and is very difficult to prove conclusively in human patients due to the multifactorial etiology and the extended prodromal phase of PD, which may span 20 years or more [1, 8, 12, 44, 50].

The mechanistic underpinnings of the hypothesis, potential risk factors, and the proposed spreading routes can be efficiently studied in animal models. However, the vast majority of previous animal studies employed intracerebral inoculation with asyn seeds, which cannot inform the dual-hit hypothesis (reviewed in [15, 49]). It has been shown that asyn aggregates can be transported retrogradely and anterogradely through the vagus [19, 53, 54]. A seminal study by Holmqvist et al. demonstrated that the preformed asyn fibrils (PFF) injected into the duodenum wall of wild-type rats were transported to the DMV within 6 days [19]. No further time points were investigated, so that finding mainly reflected the transport of the injected matter, and not the recruitment of endogenous asyn, or *trans*-synaptic propagation of asyn pathology.

Intraperitoneal and intramuscular injections of PFFs in transgenic M83 mice lead to manifest neuroinvasion of asyn pathology and in some cases, a motor phenotype [4, 11, 41, 43]. In addition, oral gavage with the insecticide rotenone in wild-type mice induces pathological asyn over time in the ENS, IML, and DMV [33, 34]. However, these peripheral seeding strategies may not be a realistic approximation of how asyn pathology actually originates and spreads in the human condition. We hypothesize that PD may start in a highly localized segment of the gastrointestinal tract, where the asyn aggregation/degradation balance is decidedly shifted toward aggregation due to the presence of local aggravating triggers such as inflammation and gut hyperpermeability or due to the stochastic formation of a particularly stable and neuroinvasive asyn strain [20, 36].

In this study, we injected PFFs in the duodenum and pylorus of a transgenic rat model to test the Braak’s hypothesis. The duodenum was chosen, as it has been shown by Holmqvist et al. that asyn injections at this site lead to robust transport to the DMV [19]. Moreover, human postmortem studies have demonstrated a gradient of asyn pathology with marked involvement of the stomach and only slight involvement of the colon [5, 17]. Also, the density of parasympathetic efferents is highest in the stomach/duodenum [7].

The transgenic rat model used in this study overexpresses the complete human SNCA gene in its wild-type form and is termed BAC (Bacterial Artificial Chromosome) in this study, as it results from the random integration of a BAC/PAC fusion construct containing the human SNCA sequence [30]. It has been shown that BAC rats overexpress cytosolic human asyn by twofold–threefold in several brain regions compared to the level of rodent asyn expressed in wild-type control rats. Thus, we expected that the excess gene dosage would significantly facilitate propagation of gut-induced PD pathology. Importantly, BAC rats display spontaneous asyn pathology only in tele- and diencephalic structures, but not in the brainstem except for the SN. Since BAC rats overexpress human asyn, we used human asyn PFFs to avoid a potential species barrier. The BAC rats were seeded in the duodenum wall with PFFs and followed for 4 months with several aims: (1) to meticulously characterize how closely the *trans*-synaptic asyn propagation follows the spreading routes predicted by the Braak staging scheme; (2) to document asyn propagation to the cardiac sympathetic nerves suggestive of anterograde propagation from the sympathetic ganglia, and (3) to document secondary involvement of the stomach, which would suggest that a primary retrograde duodenum-to-DMV propagation is followed by a secondary anterograde DMV-to-stomach propagation.

## Materials and methods

### Generation of fibrils

Two types of recombinant preformed fibrils (PFFs) were used: wild-type human asyn (phosphorylatable at Ser129 residue) and asyn S129A mutant (non-phosphorylatable at Ser129 residue). Note that both fibril types are phosphorylatable at other phosphorylation sites than Ser129. PFFs were made by incubating monomeric wild-type or S129A mutant asyn (5 mg/ml) in sterile PBS at pH 7.4 (Gibco) for 48 h at 37 °C and 1050 rpm (Thermomixer; Eppendorf). The presence of insoluble amyloid fibrillar material was confirmed by SDS-PAGE (sodium dodecyl sulfate-polyacrylamide gel electrophoresis), thioflavin T, and K114 amyloid fibril assays, as previously described [13, 56]. The SDS-PAGE of mildly denatured PFFs and monomer demonstrated that the PFFs contain a considerably higher molecular weight smear in contrast to the monomer that runs as a single 20 kDa band (Online Resource Fig. 1a). The thioflavin T fluorescence signal showed a high amyloid binding in the PFF samples (highest in the S129A), and no binding in the monomeric asyn samples, nor in the blank PBS control (Online Resource Fig. 1b). The mean K114 fluorescence signal of the fibrils is significantly higher than the mean signal of the monomeric asyn (*p* < 0.0001, Online Resource Fig. 1c). Next, we resuspended the insoluble pelleted amyloid material to 2 mg/ml in sterile PBS at pH 7.4 (Gibco) and sonicated asyn PFFs to an average size of ~ 50 nm hydrodynamic radius, as determined by dynamic light scattering (DLS) with DynaPro Star (Wyatt Technology) (25 °C). The DLS measurements of PFFs showed a homogeneous PFF population of 44 nm (Online Resource Fig. 1d). PFFs were aliquoted and stored at − 80 °C until use. The fibrils were thawed and sonicated (Branson Sonifier SFX250; Kebo Lab) for 5 min with the duty cycle setting = 30% and power output setting = 3, right before use.

In some previous studies, it cannot be excluded that the transmission of asyn pathology is caused by the simple diffusion or transport of the injected asyn material, instead of actual conversion of normal endogenous asyn to pathological asyn species. To overcome this matter, we used asyn S129A, an asyn mutant that cannot be phosphorylated at Ser129, hereby enabling us to distinguish the endogenously phosphorylated asyn from the *trans*-synaptic transportation of the exogenously injected S129A PFFs. To verify the specificity of the phospho antibodies to detect phosphorylated asyn (and not the S129A PFFs), we carried out biochemical experiments with both recombinant monomeric (pSer129) and fibrillar WT and S129A asyn run on PAGE and analyzed with one phospho-specific antibody that was used on all tissue types in this study (Ab51253) and one antibody against total asyn (Ab138501). These data are shown in Online Resource Fig. 1e. In short, PFFs or monomeric asyn (a kind gift from prof. Hilal A. Lashuel, EPFL Switzerland) were diluted in SDS loading buffer to a final concentration (50 mM Tris pH 6.8, 4% SDS, 40% glycerol, 50 mM DTE, bromophenol blue) and heated for 10 min at 95 °C. 25 ng pSer129 asyn and 100 ng PFF were added to the wells of 4–20% polyacrylamide gel (GenScript). The gel was subsequently blotted on a PVDF membrane, which was then fixed in 4% PFA for 30 min, boiled for 5 min in PBS followed by blocking in milk blocking buffer with phosphatase inhibitors (25 mM β-glycerophosphate, 1 mM Na_3_VO_4_, 5 mM NaF). Next, the membrane was incubated at 4 °C with primary antibody (Ab138501, Ab51253) and diluted in blocking buffer with phosphatase inhibitors. The membrane was then washed and incubated for 1.5 h with horseradish peroxidase conjugated secondary antibody (Dako), followed by visualization with ECL in a Fuji Las-3000 intelligent dark box.

### BAC rat model

In this study, we utilized homozygous BAC rats that are overexpressing the full-length human SNCA sequence under the control of the endogenous human regulatory elements and wild-type (Sprague–Dawley) littermates as controls. To distinguish between homozygous and heterozygous animals, the relative number of DNA copies was estimated by quantitative real-time PCR and rat tail genomic DNA. Homozygous BAC rats exhibit soft phenotype changes such as early smell deficits and late motor decline. However, these phenotype changes are irrelevant to the current research hypothesis as we are focusing on the early prodromal spread of asyn pathology through the peripheral connectomes. We refer to Nuber et al. (2013) for a detailed description of the generation of the BAC strain, genotyping, and phenotype [30].

### Animal surgery

The experimental procedures involving animals were approved by The Danish Animal Experiments Inspectorate in Glostrup, Denmark on the license 2016-15-0201-01004. The rats were acclimated for at least 1 week, and they were housed in type III cages in a Scantainer (Scanbur, Denmark). The temperature was kept at 20–24 °C and with a target humidity of approximately 45–65%. The day length was artificially held on 12 h. The rats were fed ad libitum with Altromin 1324 (Brogaarden, Denmark) and had access to tap water. Health status was controlled through regular surveys of the rodents in the facility.

Four-month-old rats (*n* = 26, 300–400 g) were randomized and anesthetized with a mixture of medical O_2_ and isoflurane (5% for induction, 2% for maintenance) and kept at a constant body temperature using a conventional heating pad. Aseptic laparotomy was performed, and the duodenum was identified. Injections of the PFFs or phosphate-buffered saline (PBS) were made using a 10 µl Hamilton syringe with a 25-gauge needle into the gut wall of the pylorus and duodenum at six different sites with an approximate spacing of 0.5 cm. Each site was injected with 3 µl of PFFs (1 µg/µl) or PBS in case of sham injections. Thirteen BAC rats and seven wild-type (WT) rats (mostly littermates) were injected with PFFs, further on referred to as ‘BACPFF’ and ‘WTPFF’, respectively. Four BAC rats and two WT rats were injected with PBS, referred to as ‘BACPBS’ and ‘WTPBS’, respectively.

Following injection, the incision was closed with several individual sutures at two levels: abdominal muscles and skin. Analgesia was administered for post-operative relief: xylocaine (2%) was applied on the skin at and around the incision site prior to surgery, buprenorphine ip (Temgesic, 2.5 mg/kg) once prior to surgery, meloxicam ip (Metacam, 0.5 mg/kg) once per day for 3 days. The rats returned to normal housing conditions and visual inspection was performed during awakening and later on a regular basis (daily during 1 week of post-operative recovery and weekly after) to assess their welfare status (including signs of neurologic disease). Soaked food was placed in the cage so the rats did not have to stretch. If the rat bed up single sutures, they were briefly anesthetized with isoflurane, after which the wound was cleaned and new sutures were placed. After surgery, the rats were continuously evaluated through a scoring scheme based on predefined human endpoints. If the score was higher than allowed in the animal license, the rat was immediately sacrificed. In that case, the rat was excluded from this study.

### Tissue collection and processing

At 2 (*n* = 11) and 4 (*n* = 15) months post-seeding, the rats were sedated and perfused transcardially with PBS and phosphate-buffered 4% formaldehyde. The brain, spinal cord, celiac ganglia, heart, stomach, and duodenum were sampled to study the route of asyn pathology spread through the ANS from the gastrointestinal tract to the brain. After overnight post-fixation, the tissue was dehydrated in a series of alcohol baths and, after clearing in xylene, embedded in paraffin. The tissue was cut into 3-µm-thick sections (Leica RM2235) and mounted on glass adhesion slides (SuperFrost Plus™, Thermo Scientific™).

### Immunohistochemistry and immunofluorescence

Tissue sections were deparaffinized in xylene and rehydrated in a series of alcohol baths. The sections were washed with TBS and 0.25% Triton X-100 in TBS (TBS-T) and incubated with a 3% hydrogen peroxide solution for 30 min to inhibit endogenous peroxidases. For antigen retrieval, slides were incubated in a modified citrate buffer (< pH 6.2) for 15 min at 80 °C. After cooling, slides were blocked with a buffer containing 2% bovine serum albumin (BSA) in TBS-T for 30 min at room temperature, and then incubated with a primary antibody diluted in 1% BSA in TBS-T at room temperature for 2–5 h. The antibodies used were: pSer129-asyn/Ab51253, pSer129-asyn/Ab59264, pSer129-asyn/A81, pSer129-asyn/Syn64; fibrillar asyn/Ab209538, total asyn/Ab138501 with or without proteinase K/Ab64220 (Abcam, 1:800) pretreatment; tubulin/Ab56676 (Abcam, 1:100); TH/Ab112 (Abcam, 1:100–1–500); VAChT/139103 (Synaptic Systems, 1:1000). Treatment with proteinase K was performed after the first washing step in TBS for 5 min at room temperature. Table 1 provides a list of the primary antibodies against asyn used in this study, including details about the supplier, concentration, and specificity.

**Table 1:** List of antibodies against alpha-synuclein

Product number	Description	Supplier	Immunogen/epitope	Concentration
Ab51253	Rabbit monoclonal [EP1536Y] to asyn (phospho S129)	Abcam	Phospho-specific peptide corresponding to residues aa 100 to the C terminus surrounding Ser129 of human asyn.	1:50–1:12500
Ab59264	Rabbit polyclonal to asyn (phospho S129)	Abcam	Phospho-specific peptide corresponding to residues surrounding Ser129 of human asyn (M-P-SP-E-E).	1:500–1:7500
A81	Mouse monoclonal to asyn (phosphor S129)	Biolegend	Phospho-specific peptide corresponding to residues 124–134 surrounding Ser129 of human asyn.	1:500–1:3000
Syn64	Mouse monoclonal to asyn (phosphor S129)	Wako	Phosphor-specific peptide corresponding to residues 124–134 surrounding Ser129 of human asyn.	1:1000
Ab209538	Rabbit monoclonal to asyn (filament conformation-spec)	Abcam	Recombinant full length protein within human asyn filament aa 100 to the C-terminus.	1:50–1:500
Ab138501	Rabbit monoclonal [MJFR1] to asyn	Abcam	Recombinant full length protein corresponding to residues 118–123 of human asyn.	1:500–1:10000

After incubation with primary antibody, the sections were rinsed and incubated with a biotinylated secondary antibody (BA-2000 or BA-2001, Vector Laboratories, 1:200) in combination with the Vectastain ABC peroxidase kit, or an HRP secondary antibody (P0448, Dako, 1:400) for 2 h. All incubation steps were done in a humidified chamber. Peroxidase-positive structures were then visualized by incubation with DAB (3,3′-diaminobenzidine) containing 0.03% H_2_O_2_ for 0.5–5 min, depending on the antibody. After counterstaining with toluidine blue, the slides were coverslipped with Eukitt mounting medium (Sigma, 03989).

Samples from each experimental group (i.e., BACPFF, BACPBS, WTPFF, WTPBS) were stained together. Additionally, positive (known presence of pathology) and negative controls (omitting the primary antibody) were included in each staining.

Triple immunofluorescence staining was performed on the duodenal tissue for co-localization purposes. The staining protocol was similar to the immunohistochemistry protocol described above, but with goat serum as the blocking agent and the primary antibodies beta-Tubulin 3/Ab78078 (Abcam, 1:500) and pSer129/Ab51253 (Abcam, 1:2000). After overnight incubation with primary antibody at 4 °C, the sections were incubated overnight at 4 °C with a secondary antibody AlexaFluor488/Ab150117 or Biotin/Ab97049 (Abcam, 1:1000). Next, the sections were incubated in Streptavidin-AlexaFluor647/405237 (Biolegend, 1:1000) and DAPI/D3571 (Invitrogen, 1:4000) for 1 h at room temperature. In between each incubation step, the sections were washed three times for at least 30 min per washing step. Finally, the sections were dehydrated and mounted with Vectashield Antifade Mounting Medium (Vector Laboratories, H-1000). Immunofluorescent images were acquired with Zeiss Confocal LSM700.

### Quantification assessment of asyn immunoreactivity

For quantification, optical density values were calculated using ImageJ (https://fiji.sc/, open-source platform for biological image analysis). Images for optical density analysis were collected from 3-μm-thick sections using the Visiopharm software (Visiopharm, Hoersholm, Denmark) and Olympus VS120 automated slide scanning microscope (Olympus, Ballerup, Denmark) with automated calibration and constant light source.

First, a background subtraction was performed based on the mean value of an unstained portion of the section, such as the corpus callosum, cerebellum, or abdominal aorta. Second, a color deconvolution (after double staining with toluidine blue) was done. Lastly, the regions of interests were drawn as shown in Fig. 2, and optical density values were calculated [40]. For statistical analysis, a one-way analysis of variance (ANOVA) with Tukey post hoc test was used for multiple comparisons among groups.

## Results

### Characterization of asyn pathology in the BAC rat brain

**Fig. 1:**
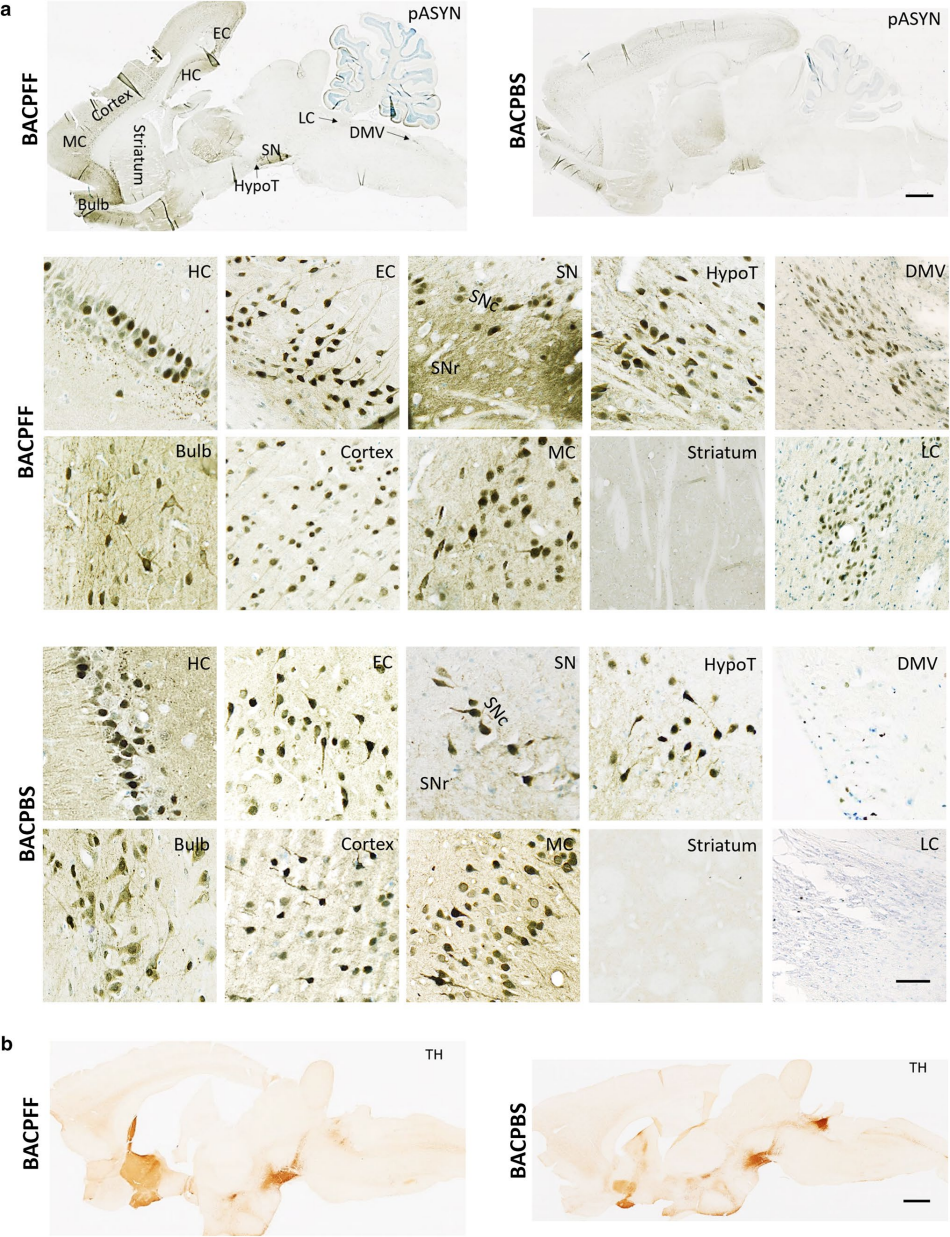
**a** Distribution of phosphorylated asyn (pASYN/Ab51253) pathology in sagittal brain sections of S129A PFF- and PBS-injected BAC rats at 4 months post-injection. The scale bar represents 1 mm in the whole brain sagittal sections (upper panel). In the lower panel, representative high-magnification photomicrographs of phosphorylated asyn (pASYN, Ab51253) pathology are shown in several brain areas of S129A PFF- and PBS-injected BAC rats: bulb, cortex, motor cortex (MC), striatum, hippocampus (HC), entorhinal cortex (EC), substantia nigra pars compacta (SNc), substantia nigra pars reticulata (SNr) hypothalamus (HypoT), dorsal motor nucleus of the vagus nerve (DMV), locus coeruleus (LC). The scale bar represents 100 µm in the DMV and LC, and 50 µm in all other brain areas. Similar levels of brain pathology were seen in the BAPFF and BACPBS rats, except for the brainstem (DMV, LC, and SNr), which contained significantly less or no pathology in the BACPBS rats. **b** Distribution of tyrosine hydroxylase in a sagittal brain section of BACPFF and BACPBS rats. Scale bar: 1 mm

**Fig. 2:**
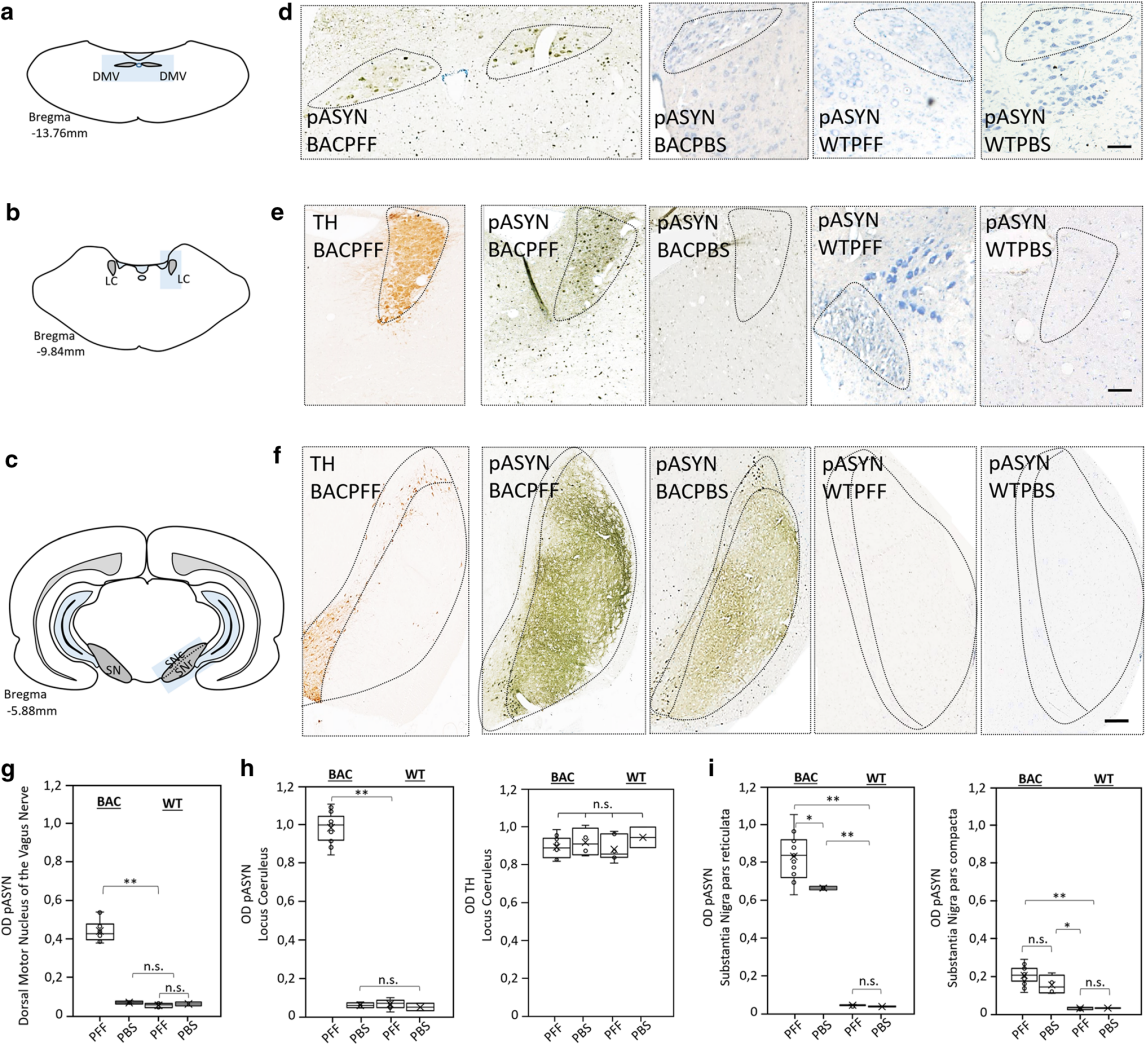
Phosphorylated asyn (pASYN) pathology in the DMV, LC, and SN of S129A PFF- and PBS-injected BAC rats and WT controls at 4 months post-injection. **a–c** Schematic of DMV (**a**), LC (**b**), and SN (**c**) anatomy at bregma − 13.76 mm, − 9.84 mm and − 5.88 mm, respectively. **d** High magnification photomicrograph of asyn pathology (pASYN/Ab51253) in the DMV of PFF- and PBS-injected BAC and WT rats. Scale bar: 200 µm. **e**, **f** High-magnification photomicrograph of tyrosine hydroxylase and asyn pathology (pASYN/Ab51253) in the LC (**e**) and SN (**f**) of PFF- and PBS-injected BAC and WT rats. Scalebar: 200 µm. **g** Optical density (OD) measurements of pASYN pathology in the DMV of PFF- and PBS-injected BAC and WT rats. The regions of interest used for the analysis are indicated in **d** and in Online Resource Fig. 6. **h** Optical density measurements of pASYN pathology (left panel) and TH (right panel) in the LC of PFF- and PBS-injected BAC and WT rats. The regions of interest used for the analysis are indicated in **e** and in Online Resource Fig. 6. The BACPFF rats showed pathology in the DMV and LC at 2 and 4 months post-injection (DMV: *p *< 0.01, LC: *p *< 0.001). The DMV and LC of BACPBS and WT rats remained free of pathology. **i** Optical density measurements of pASYN pathology in the SN pars reticulata (left panel) and pars compacta (right panel) of PFF- and PBS-injected BAC and WT rats. The regions of interest for the analysis are indicated in **f** and in Online Resource Fig. 6. The BACPFF rats showed significantly more pathology in the SNr (*p *< 0.05) at 2 and 4 months post-injection, but not in the SNc. The SN of WT rats remained free of pathology. Each data point represents mean OD values. Horizontal bars: ± SE

**Fig. 3:**
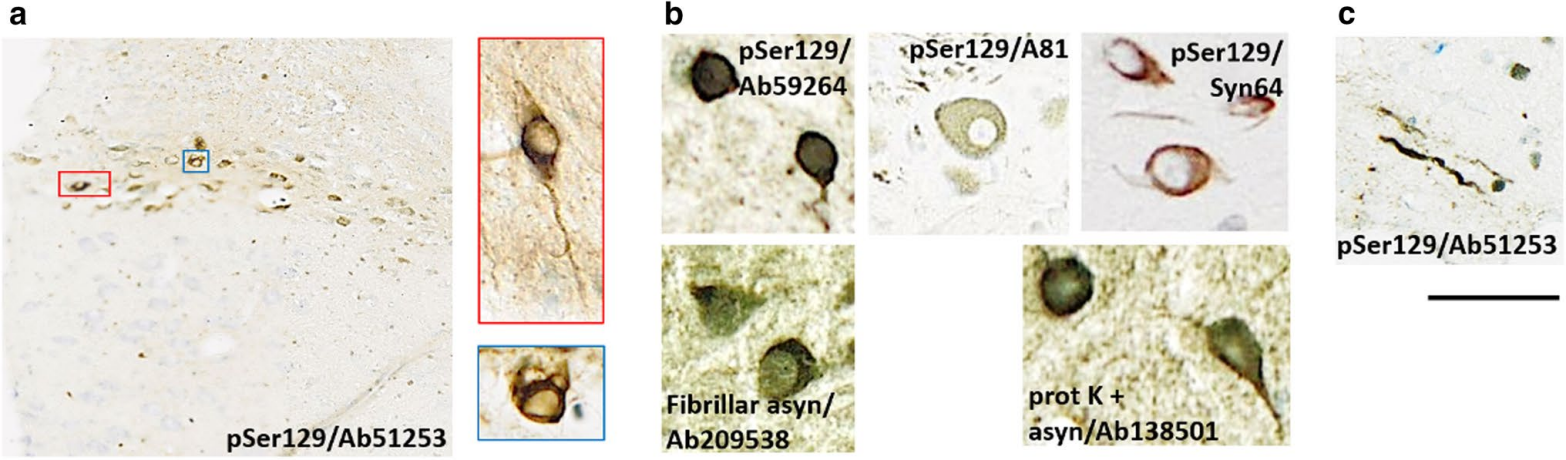
Representative high magnification photomicrographs of phosphorylated asyn-positive inclusions detected in the DMV of seeded BAC rats. Inclusions were evident in neuronal cell bodies (**a**, **b**) and as elongated Lewy neurite-like structures (**c**). Depending on the antibodies used, inclusions (a/b, scale bar = 25 µm) and neurites (**c**, scale bar = 50 µm) had different appearances

At 4 months post-injection, we detected in PFF- and PBS-injected BAC rats alike marked asyn immunoreactivity in the olfactory bulb, motor, frontal, parietal, occipital, and entorhinal cortex, striatum, thalamus, hippocampus, hypothalamus, and the substantia nigra (SN). However, the BACPFF rats showed more intense asyn staining in the SN pars reticulata (SNr) than the BACPBS rats, and only the BACPFF rats exhibited distinct asyn pathology in the dorsal motor nucleus of the vagus (DMV) and the locus coeruleus (LC). In contrast, the brainstem of all BACPBS rats remained completely free of pathology below the level of the SN. No asyn pathology was seen anywhere in the brain of all WT rats. We refer to Fig. 1a and Online Resource Figs. 2, 3, 4, and 5 for representative images of the ‘whole-brain´ asyn pathology distribution in sagittal brain sections of seeded and non-seeded BAC and WT rats, detected with six different antibodies in total. Figure 1b shows the tyrosine hydroxylase (TH) distribution in a sagittal brain section of a BACPFF and a BACPBS rats. There was no difference in the level or pattern of TH between all four groups. Figure 2 shows representative images from brainstem structures (DMV, LC, and SN) of BAC and WT rats and the optical density analyses (full dataset in Online Resource Fig. 6). Significantly more asyn pathology was detected in the BACPFF group in the DMV (*p* < 0.01), LC (*p* < 0.001), and SNr (*p* < 0.05), but not in the SN pars compacta (SNc), compared to the other experimental groups. Figure 3 shows representative magnifications of asyn inclusions in the DMV and LC of the seeded BAC rat, as detected by several antibodies. The detection of asyn aggregates, which are phosphorylated at Ser129 after duodenal seeding with S129A fibrils, indicates recruitment of endogenous asyn to pathological aggregates in the recipient cell, rather than the mere transport of the injected fibrils.

### Primary parasympathetic and sympathetic retrograde propagation from the ENS to the brain

**Fig. 4:**
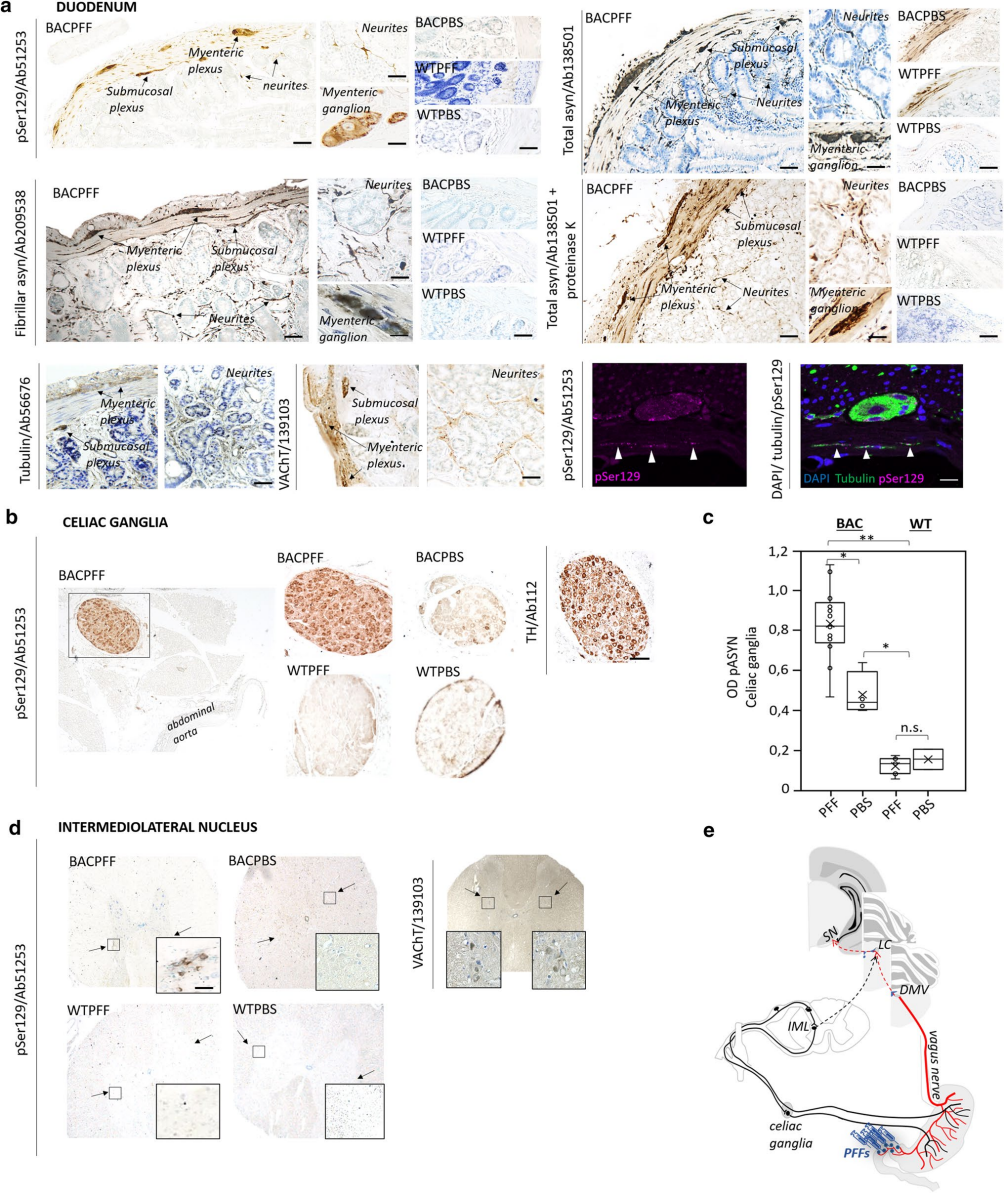
**a** Distribution of asyn pathology in the duodenum of S129A PFF- and PBS -injected BAC rats and WT controls, at 4 months post-injection, detected with four different antibodies. Scale bar: 100 µm. Representative high magnification photomicrographs are shown of the myenteric ganglion and neurites in the BACPFF. The enteric nervous system remained pathology free in BACPBS and WT rats. Scale bar: 50 µm. For co-localization purposes, representative high magnification photomicrographs are shown of the distribution of VAChT, tubulin, and DAPI in the duodenum. Asyn pathology co-localized with DAPI, VAChT, and tubulin. Scale bar: 50 µm. **b** Distribution of asyn pathology in the celiac ganglia of S129A PFF- and PBS-injected BAC rats and WT controls, at 4 months post-injection (left panel). Distribution of tyrosine hydroxylase in the celiac ganglion of a BAC PFF rat (right panel). The TH distribution is similar across the four experimental groups. Scale bar: 200 µm. **c** Optical density (OD) measurements of pASYN pathology in the celiac ganglia of PFF- and PBS-injected BAC and WT rats. pASYN levels were significantly higher in the ganglia of BACPFF compared to BACPBS rats (*p *< 0.05), and were close to zero in the WT rats. **d** Distribution of asyn pathology in the IML of S129A PFF- and PBS-injected BAC rats and WT controls, at 2 or 4 months post-injection (left panel). For co-localization purposes, distribution of VAChT in the IML of a BAC PFF rat. The VAChT distribution is similar across the four experimental groups (right panel). High magnification photomicrographs of the IML are shown in the right bottom corner of each image. 4/13 BACPFF showed unilateral asyn pathology and 9/13 BACPFF showed bilateral asyn pathology in the IML after duodenal seeding. No pathology was observed in the IML of BACPBS and WT rats (see Table 2 and Online Resourse Fig. 9). Scale bar: 50 µm. **e** Schematic of the proposed retrograde parasympathetic (duodenum → DMV → LC) and sympathetic (duodenum → celiac ganglia → IML → LC) spreading routes of asyn pathology

Along the parasympathetic pathway, asyn pathology propagated from the duodenum to the DMV, and along the sympathetic pathway to the celiac ganglia, and then to the intermediolateral nucleus of the spinal cord (IML). Figure 4 depicts pathological asyn in the duodenum, autonomic ganglia, and IML of BACPFF rats (full data set in Online Resource Figs. 7, 8, and 9). No asyn pathology was seen anywhere in the ENS, autonomic nuclei, or IML in the seeded or non-seeded WT rats. Some phosphorylated asyn pathology was observed in the duodenal ENS of the BACPBS group. However, this pathology tended to be in the cell nuclei and disappeared almost completely when using a proteinase K pretreatment before staining against phosphorylated asyn. In contrary, the BACPBS rats showed some heterogeneous staining in a few cell bodies of the celiac ganglia even after using proteinase K pretreatment. Thus, the BACPBS rats might display some spontaneous pathology in the periphery, but it was clearly below the levels seen in BACPFF rats (*p* < 0.05). Asyn pathology was seen to co-localize with several markers: DAPI, microtubulin, and the vesicular acetylcholine transporter (VAChT) in the duodenum (Fig. 4a), tyrosine hydroxylase in the celiac ganglia (Fig. 4b), and VAChT in the IML (Fig. 4d). The pathology in the myenteric and submucosal plexuses and the neurites in the mucosa resembled pathological asyn immunoreactivity in the colon of a human PD patient (Online Resource Fig. 10).

### Secondary anterograde propagation to the heart and stomach

**Fig. 5:**
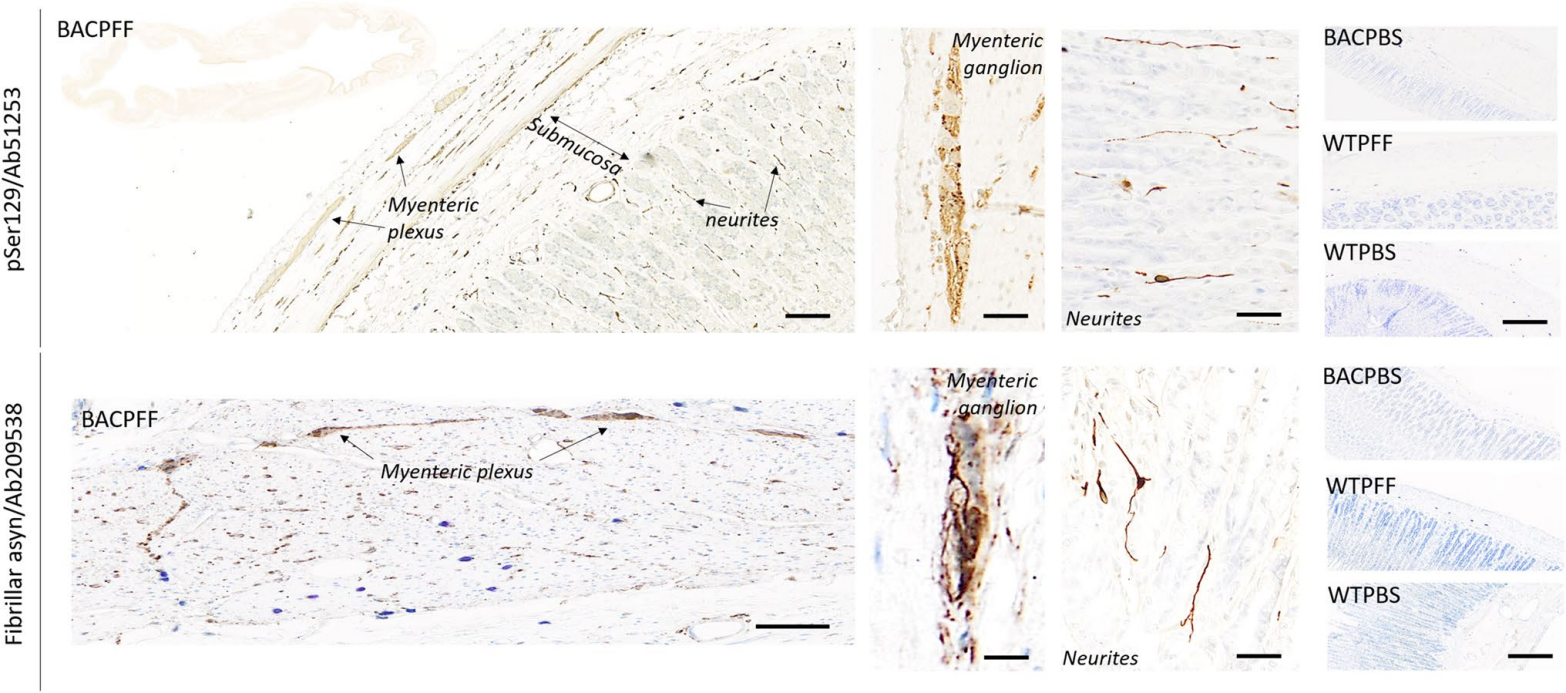
Distribution of asyn pathology in the stomach of S129A PFF- and PBS-injected BAC rats and WT controls, at 4 months post-injection, detected with two different antibodies. Scale bar = 100 µm. Representative high magnification photomicrographs are shown of the myenteric ganglion and neurites in the BACPFF. The plexus and lumen of the BACPBS and WT rats remained pathology free. Scale bar: 50 µm

**Fig. 6:**
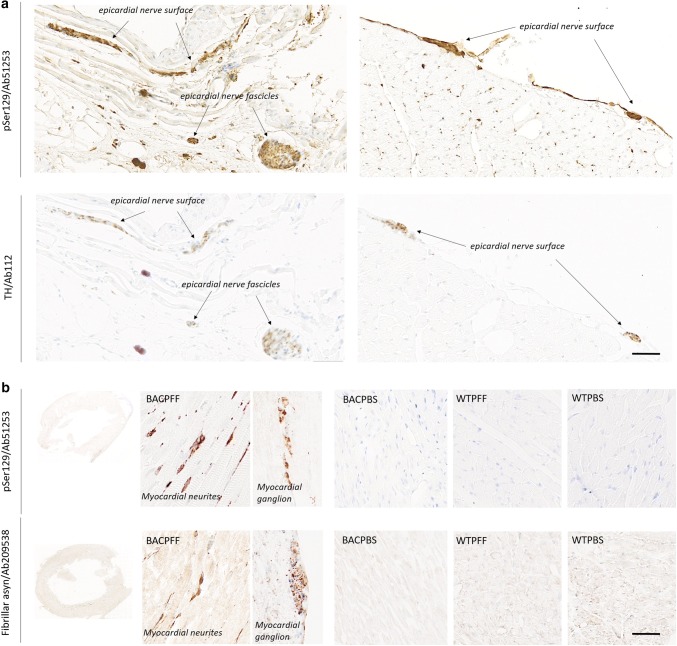
**a** Distribution of asyn pathology (pSer129/Ab51253; upper panel) and tyrosine hydroxylase (lower panel) in the heart of S129A PFF- and PBS-injected BAC rats and WT controls at 4 months post-injection. In the BACPFF rats, clear co-localization was seen between staining for TH and phosphorylated asyn pathology. Scale bar = 100 µm. **b** Distribution of asyn pathology in the myocardium of S129A PFF- and PBS-injected BAC rats and WT controls, detected with two different antibodies. Representative high magnification photomicrographs are shown of a myocardial ganglion and neurites in the BACPFF. The heart remained free of pathology in all BACPBS and WT rats. Scale bar: 50 µm

Marked asyn pathology was detected in the stomach (Fig. 5) and the heart (Fig. 6) of BACPFF rats (full dataset in Online Resource Figs. 11, 12). In the heart, the asyn signal co-localized with TH staining (Fig. 6a). These findings suggest secondary anterograde propagation from the DMV to the stomach and from the autonomic ganglia and sympathetic trunk to the cardiac sympathetic nerves after the initial retrograde propagation along the parasympathetic and sympathetic pathways.

**Fig. 7:**
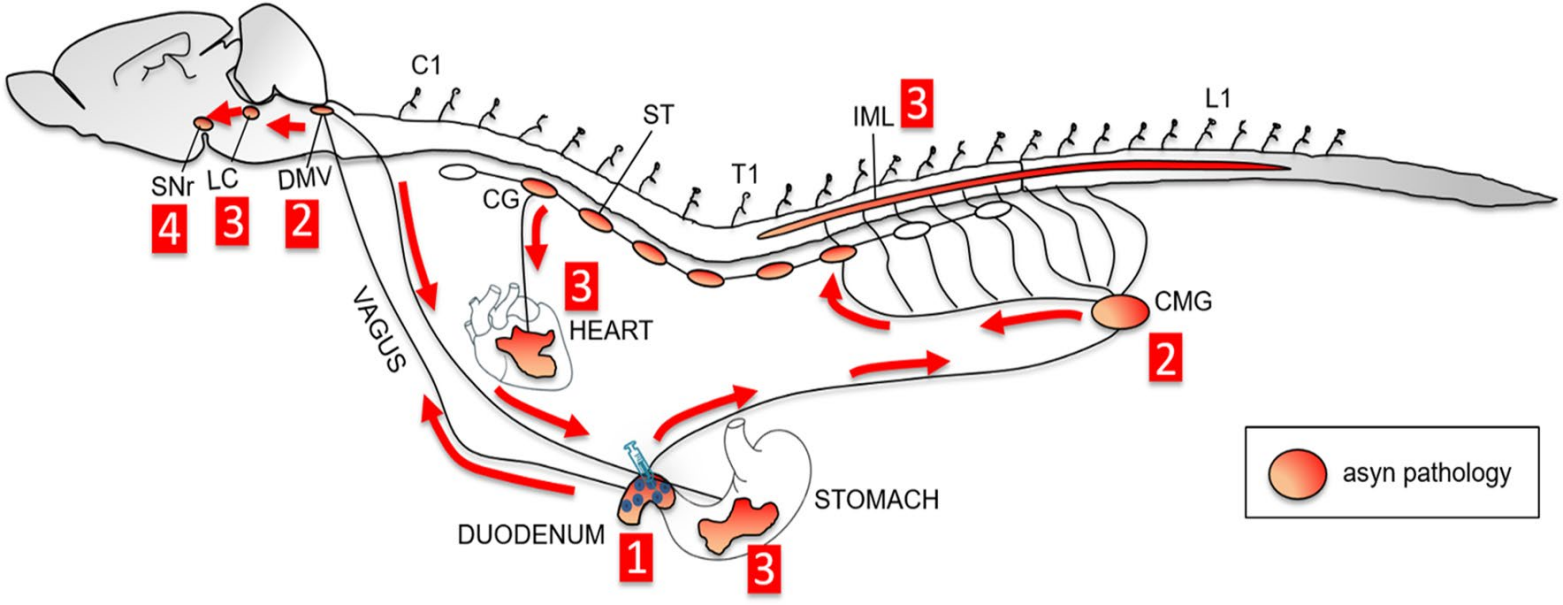
Schematic overview of the hypothesized *trans*-synaptic bidirectional propagation of asyn pathology through the autonomic nervous system to the brain after gastrointestinal seeding. First, seeding of the duodenum with fibrils induces aggregation of endogenous asyn in the duodenum. Second, the induced pathology retrogradely spreads along the parasympathetic pathway to the dorsal motor nucleus of the vagus (DMV) and along the sympathetic pathway to the celiac ganglia (CMG). Third, asyn pathology propagates *trans*-synaptically to the locus coeruleus (LC) and intermediolateral nucleus of the spinal cord (IML). Additionally, asyn pathology spreads anterogradely to the heart and to the stomach. Fourth, asyn pathology spreads to the substantia nigra pars reticulata (SNr)

Figure 7 depicts a schematic overview of the proposed *trans*-synaptic and bidirectional propagation of asyn pathology, and Table 2 summarizes the frequency of asyn pathology in the analyzed CNS and ANS tissues in BACPFF rats. Some intersubject variability in disease progression was seen in the heart and IML, which may indicate temporal evolution of the propagating pathology. 2 months post-seeding, asyn pathology was detected in the bilateral IML (BiL-IML) in 3/5 and unilateral IML (UniL-IML) in 2/5 BACPFF rats, and in the heart in 2/5 BACPFF rats. 4 months post-seeding, asyn pathology was detected in the BiL-IML in 6/8 and UniL-IML in 2/8 BACPFF rats, and in the heart in 7/8 BACPFF rats. The intersubject variability is also evident from the Online Resource figures, which provide the full dataset per tissue type analyzed (subjects seeded with S129A PFFs are marked with * in the label).

**Table 2:** Presence of asyn pathology in BAC rats after seeding with PFF

No. of seeded BAC rats affected								
mpi	Duodenum	DMV	Celiac ganglia	LC	Stomach	Heart	UniL-IML	BiL-IML
2	5/5	5/5	5/5	5/5	4/5	2/5	2/5	3/5
4	8/8	8/8	8/8	8/8	8/8	7/8	2/8	6/8

*mpi* months post-injection, *UniL*-*IML and BiL*-*IML* unilateral and bilateral intermediolateral nucleus

## Discussion

Here, we injected human wild-type or mutant asyn fibrils, or vehicle, in the duodenum wall of homozygous BAC rats and WT littermates. To investigate the early spread of asyn pathology, the tissue was collected at 2 and 4 months post-seeding. Our study provides the hitherto most comprehensive immunohistochemical report about propagation of initially localized enteric asyn pathology through the autonomic nervous system to the brain in a transgenic rodent model. We documented *trans*-synaptic propagation of phosphorylated asyn pathology through interconnected neuronal pathways in a pattern, which fully recapitulates Braak’s hypothesis.

Our study design was similar to the seminal study by Holmqvist et al. [19]. However, the observational period in that study was restricted to 6 days, which is much too short to document formation of pathological asyn aggregates through recruitment of endogenous asyn. Furthermore, the investigators did not use antibodies against pathological, phosphorylated asyn, but only a single antibody directed against total human asyn without proteinase K pretreatment. That study was, therefore, limited to detect only short-term vagal transport of the injected asyn aggregates to the DMV.

### Gut-to-CNS propagation of asyn pathology

In the present study, duodenal injections of PFFs were followed by unequivocal asyn pathology in the ENS, DMV, celiac ganglion (CMG), and LC in all 13 BAC rats. Markedly, less asyn pathology was seen in the IML, with 9/13 BAC rats displaying pathology bilaterally and 4/13 only unilaterally. In contrast, none of the rats in the other experimental groups displayed clear asyn pathology in any of these structures (after proteinase K pretreatment of the tissue), except in the CMG where we did see slightly more phosphorylated asyn staining in the BACPBS group compared to the clearly negative WT rats (Fig. 4b). Thus, it is possible that the BAC rat model also shows subtle spontaneous asyn pathology in the CMG in addition to the much more dramatic pathology in the telencephalon. Interestingly, the sympathetic chain and autonomic ganglia may be particularly susceptible to asyn pathology, since incidental Lewy body disease patients tend to show the highest frequency of asyn pathology in these structures [5, 17]. Nevertheless, the CMG staining in the BACPFF group was significantly more positive—suggestive of a significant contribution from propagating asyn pathology initiated by the injected PFFs.

In the SN of the BACPBS rats, the magnitude of asyn staining was generally uniform across the SNc and SNr, whereas the SNr appeared more intensely stained in the BACPFF rats (Online Resource Fig. 6). This qualitative impression was supported by the optic density measurements (Fig. 2i). Since it has been reported that the LC projects mainly to the SNr [6, 55], it seems plausible that the additional asyn pathology of the SNr was caused by asyn propagation through LC connections. Though neuronal connectivity is necessary, additional factors might contribute to the anatomical pattern of asyn pathology propagation [32]. Overall, our findings suggest that seeded BAC rats show asyn propagation through Braak stages 1, 2, and 3 [9, 10].

The present finding of robust and extensive gut-to-brain asyn propagation contrasts with two recent reports [25, 52]. In those studies, asyn PFFs were injected directly into the stomach or colon of mice, rats, and macaques, but little or no persisting asyn pathology was seen in the brainstem. Importantly, those investigators employed young healthy wild-type animals without additional susceptibility factors. The seminal paper by Holmqvist et al. reported asyn positivity in the DMV at 6 days, which most likely represent staining of the transported injected material. It seems probable that this asyn signal would also have become negative, had the authors included time points beyond 6 days [19]. We would argue that young wild-type animals (including humans) are probably able to minimize or prevent neuroinvasion from sites of enteric asyn aggregation, given that sporadic PD is mainly diagnosed in people above 60–70 years of age [16]. Therefore, the use of young wild-type animals could lead to false- negative results within this experimental paradigm. Surprisingly, the effects of aging on the efficiency of asyn propagation in animal models have received almost no attention so far.

Of note, we did not see asyn pathology in the myenteric and submucosal plexus of the PFF-injected WT rats, which contrasts with the observed persistent enteric pathology in PFF-injected WT rodents and non-human primates [25, 52]. The absence of pathology in the present WT rats could be explained by a species barrier, since we used human asyn PFFs in the WT rats, whereas the previous studies used species-compatible PFFs.

### Potential bidirectional spread

To our knowledge, this study is the first to investigate the potential secondary anterograde (DMV-to-stomach) spreading of asyn pathology, after the primary retrograde (duodenum-to-DMV) spreading within the same study protocol. We detected asyn pathology in the stomach in 4/5 rats at 2 months, and 8/8 rats at 4 months post-injection. The pathology was found 3 cm or more from the site of duodenal injection. During PFF injections, care was taken not to penetrate into the duodenal lumen or veins. The needle was slowly pushed horizontally into the wall for at least 1 cm and left in place for at least 15 s after injection. Upon retraction, the injection site was carefully inspected and dabbed clean to remove any invisible amount of leaking fluid to the peritoneal space. Thus, we believe that only negligible amounts of PFFs could have escaped to the peritoneum. To our knowledge, no long distance ENS connections have been demonstrated between the duodenum and stomach. Since the DMV and the celiac ganglion were asyn-positive in all rats at 2 months post-injection, the pathology in the stomach could conceivably have traveled to the stomach via both the sympathetic and parasympathetic pathways. However, it cannot be excluded that the stomach pathology was caused by accidental seeding from PFFs escaped from the injection site, or that asyn pathology propagates rapidly through the ENS connectome. The latter seems improbable, since the pathology would likely have to cross a large number of synapses.

Future studies could more conclusively prove that such widespread enteric pathology is caused by bidirectional asyn propagation through the vagus by performing similar experiments in vagotomized rats. Also, a decreasing density of asyn pathology in the myenteric plexus from the stomach to the transverse colon would suggest that the pathology was derived from DMV motor efferents, which are known to exhibit this pattern of innervation [7]. Of note, the majority of asyn-positive synapses in the rat myenteric plexus are DMV motor efferents, whereas sensory vagal afferents are asyn-negative and local enteric neurons express asyn at much lower frequencies [37, 46, 47, 54]. Thus, the DMV motor efferents could be a particular vulnerable system for asyn pathology and propagation.

The decreasing gradient of asyn pathology has also been demonstrated in whole body autopsy studies of human PD patients [5, 17]. If the present observation of secondary stomach pathology was caused by bidirectional propagation through the vagus, it could have significant importance for our understanding of PD. It has been suggested that the relative absence of human postmortem cases with “gut-only” asyn pathology argues against the dual-hit hypothesis [3]. However, the present findings suggest that the origin of PD could be highly localized enteric pathology, which rapidly spreads to the DMV and then back to the entire projection field of DMV motor neurons. Detecting the localized initial pathology in the human gastrointestinal tract, which measures more than 8–10 m in length, would be extremely challenging unless hundreds of tissue samples were examined throughout the gut. In addition, the time window from the appearance of initial enteric pathology to the appearance of the first DMV inclusion may be very short (weeks). Thus, the absent gut-only cases in human autopsy studies cannot presently be considered proof that PD does not start in the gut. Rather, those data suggest that, if PD starts in the gut in some cases, the initial enteric pathology is probably very localized.

### Heart pathology

Severe degeneration of the cardiac postganglionic sympathetic projections is a well-known characteristic of PD, and by Hoehn and Yahr stage 3, nearly all PD patients exhibit manifest cardiac denervation [21, 29]. Moreover, nearly all prodromal PD patients with REM sleep behavior disorder display fulminant cardiac denervation at an early time point, when their nigrostriatal dopamine system is still mostly intact [22, 28]. The etiology behind cardiac denervation is unknown, but it seems clear that the noradrenergic postganglionic neurons also constitute a particularly vulnerable cell population.

We hypothesized that initial asyn pathology located in the gut could rapidly propagate via the celiac and stellate ganglia to the cardiac sympathetic nerve terminals, and our findings support this hypothesis. Two of five rats showed pathology at 2 months, whereas 7/8 rats were positive at 4 months post-injection. Our histological findings coincide with previous studies of asyn pathology and sympathetic innervation in the heart [31, 57]. Of note, the pattern of positive asyn staining as well as identifiable TH staining in the BACPFF rats is compatible with findings in ILBD patients. Once cardiac neurodegeneration is underway in manifest PD, both of these signals disappear [31]. The asyn signal disappears because there are very few sympathetic terminals left to harbor asyn pathology.

Overall, our observations demonstrate that phosphorylated asyn pathology in the heart of PD patients could be a secondary phenomenon to initial onset in the gut, and that it takes only 2–4 months for cardiac pathology to develop in rats. Although humans are considerably larger mammals than rats, the prodromal phase of PD probably spans 20 years or more in many patients, which would be a sufficient time for the asyn propagation to occur with deleterious consequences for the vulnerable sympathetic cardiac nerves. The current findings, therefore, present a plausible explanation for the conundrum of cardiac denervation in prodromal PD. Importantly this explanation is fully compatible with the dual-hit hypothesis, since the heart pathology was only seen in the gut-seeded BAC rats.

### Limitations

No animal models faithfully recapitulate all pathological and clinical features of PD. The BAC rat could be construed as a model of genetic PD with SNCA multiplication and the present findings may, therefore, not be applicable to normal sporadic PD. On the other hand, transgenic models such as the BAC rat could be a general approximation of unbalanced asyn aggregation/degradation, and may, therefore, be applicable to situations with normal amounts of asyn, but decreased cellular clearance mechanisms, which may be a consequence of normal aging. Of note, very little is known about asyn propagation in aged wild-type animals.

As mentioned above, the observed asyn pathology in the stomach could be a consequence of peritoneal seeding from escaped PFFs at the injection site or tangential propagation through the ENS. Both the cardiac and stomach asyn pathology could theoretically also have been caused by hematogenic seeding. Considering the striking asyn pathology in the DMV and celiac ganglion in all rats at 2 months post-injection, we find these alternative explanations less likely, but future studies could investigate such spreading routes using vagotomized animals, and by performing intravenous injections of PFFs.

Finally, it has been reported that cross seeding between human and mouse asyn is less efficient than homologous seeding [24]. Therefore, it cannot be excluded that mismatches in the sequence between human asyn PFFs and wild-type rat asyn could have impeded seeding in the WTPFF group of this study. However, Abdelmotilib et al. did not observe any difference in seeding effectiveness of human and mouse asyn fibrils in mouse primary neurons [2], and both human and mouse asyn fibrils have been used successfully in different seeding studies [24, 26, 27, 35, 42]. Moreover, Recasens et al. have shown that intracerebral seeding of wild-type mice with human PD lysate induces asyn pathology and progressive nigral degeneration 3–4 months post-seeding, indicating that exogenous human asyn can be quickly internalized and trigger asyn pathology in mice [38]. Different strains, compositions, and concentrations of fibrils have been used in different animal models across studies, but it remains incompletely understood how these factors contribute to seeding efficiency. A comparative study in vivo is necessary to elucidate the seeding efficiency across animal models.

## Conclusion

In summary, we have provided conclusive evidence that duodenal seeding with preformed asyn fibrils in transgenic BAC rats leads to robust *trans*-synaptic asyn propagation of endogenously recruited asyn in a pattern fully compatible with the “body-first hypothesis” of PD etiopathogenesis. Propagation was documented through the vagus to the DMV, and through the sympathetic connectome to the celiac ganglion and IML. The pathology propagated rostrally in the brainstem with involvement of the LC and SNr. We also provide the first evidence of involvement of the cardiac sympathetic nerves, which most likely signifies rapid propagation via the sympathetic trunk. This finding provides an explanation for the well-known observation that cardiac denervation occurs in the prodromal phase of PD. In addition, we observed secondary asyn pathology in the stomach. We speculate that localized gastrointestinal asyn pathology initially spreads to the DMV, infects neighboring cell bodies, and then spreads anterogradely via DMV motor efferents. This proposal reconciles the gut-first hypothesis with the observed rostro-caudal distribution of asyn pathology in autopsy studies of PD patients. The BAC rat model could be very valuable for detailed mechanistic studies of the dual-hit hypothesis, and for studies of disease modifying therapies targeting early pathology in the gastrointestinal tract.

## Electronic supplementary material

Below is the link to the electronic supplementary material.

Supplementary material 1 (PDF 2930 kb)
