# Supplementary material for: Evidence for bidirectional and trans-synaptic parasympathetic and sympathetic propagation of alpha-synuclein in rats
Source: Acta Neuropathol. 2019 Jun 26;138(4):535–50. doi: 10.1007/s00401-019-02040-w (PMC6778265; doi:10.1007/s00401-019-02040-w)
Supplement: Supplementary file 1 — Supplementary material 1 (PDF 2930 kb) [file 401_2019_2040_MOESM1_ESM.pdf]

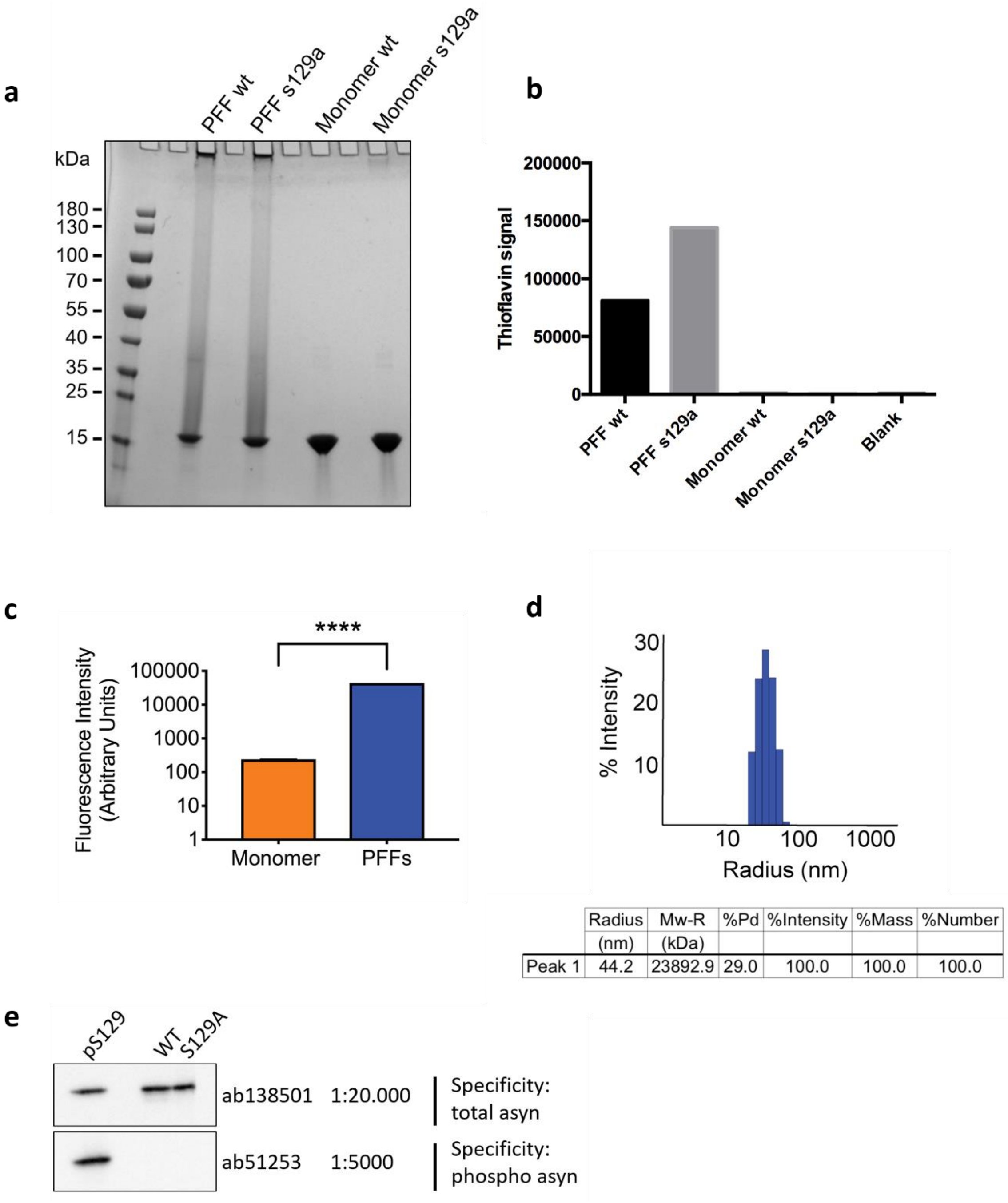

	Radius (nm)	Mw-R (kDa)	%Pd	%Intensity	%Mass	%Number
Peak 1	44.2	23892.9	29.0	100.0	100.0	100.0

Online Resource Fig. 1 Asyn fibril formation and biochemical characterization. Soluble monomeric S129A asyn or WT asyn (5mg/ml) was aggregated into preformed fibrils (PFFs) by incubation at 37°C in PBS, pH 7.4, with continuous shaking at 1050 rpm for 48 h. Samples were spun down and equal volumes of asyn proteins in the pellets (P-fraction, insoluble fibrillar material) and supernatants (S-fraction, soluble non-fibrillar material) were then subjected to (**a**) SDS-PAGE analysis (20µg/lane), and visualized by Coomasie staining. (**b**) Thioflavin T signal of PFF and monomers of WT or S129A asyn. A blank control of PBS was also included. Emission intensity at 482nm was measured, after excitation at 450nm. (**c**) K114 fluorescent amyloid fibril binding assay of asyn monomers (negative control) and PFFs (P-fraction) ($p<0.0001$). (**d**) Dynamic Light Scattering (DLS) analysis of PFFs demonstrating a homogeneous PFF population of 44nm. (**e**) Fibrils (WT/S129A) were denatured and loaded on SDS-PAGE alongside control phosphorylated synuclein (pS129). Proteins were then immunoblotted, and visualized using a primary antibodiy against total (Ab138501) and phosphorylated (Ab51253) asyn

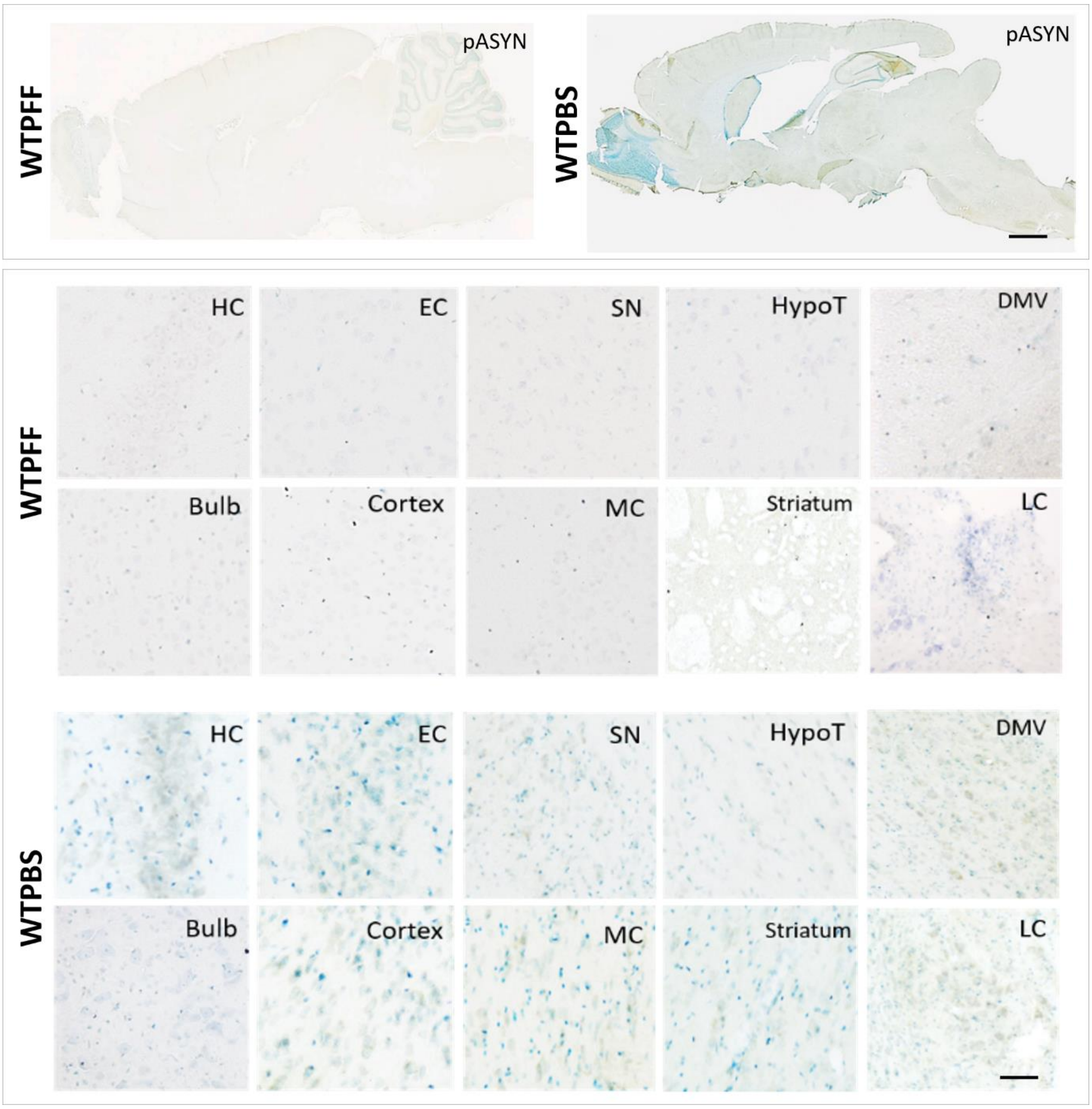

Online Resource Fig. 2 No phosphorylated asyn (pSer129/Ab51253) pathology was detected anywhere in the brain of PFF- and PBS-injected WT rats at four months post injection. Upper panel shows sagittal brain sections of both WT groups. The scale bar represents 1 mm in these whole brain sagittal sections. In the lower panel, representative high-magnification photomicrographs of relevant brain structures shown from the PFF- and PBS-injected WT rats: bulb, cortex, motor cortex (MC), striatum, hippocampus (HC), entorhinal cortex (EC), substantia nigra (SN), hypothalamus (HypoT), dorsal motor nucleus of the vagus nerve (DMV), locus coeruleus (LC). The scale bar represents 100 µm in the DMV and LC, and 50 µm in all other brain areas

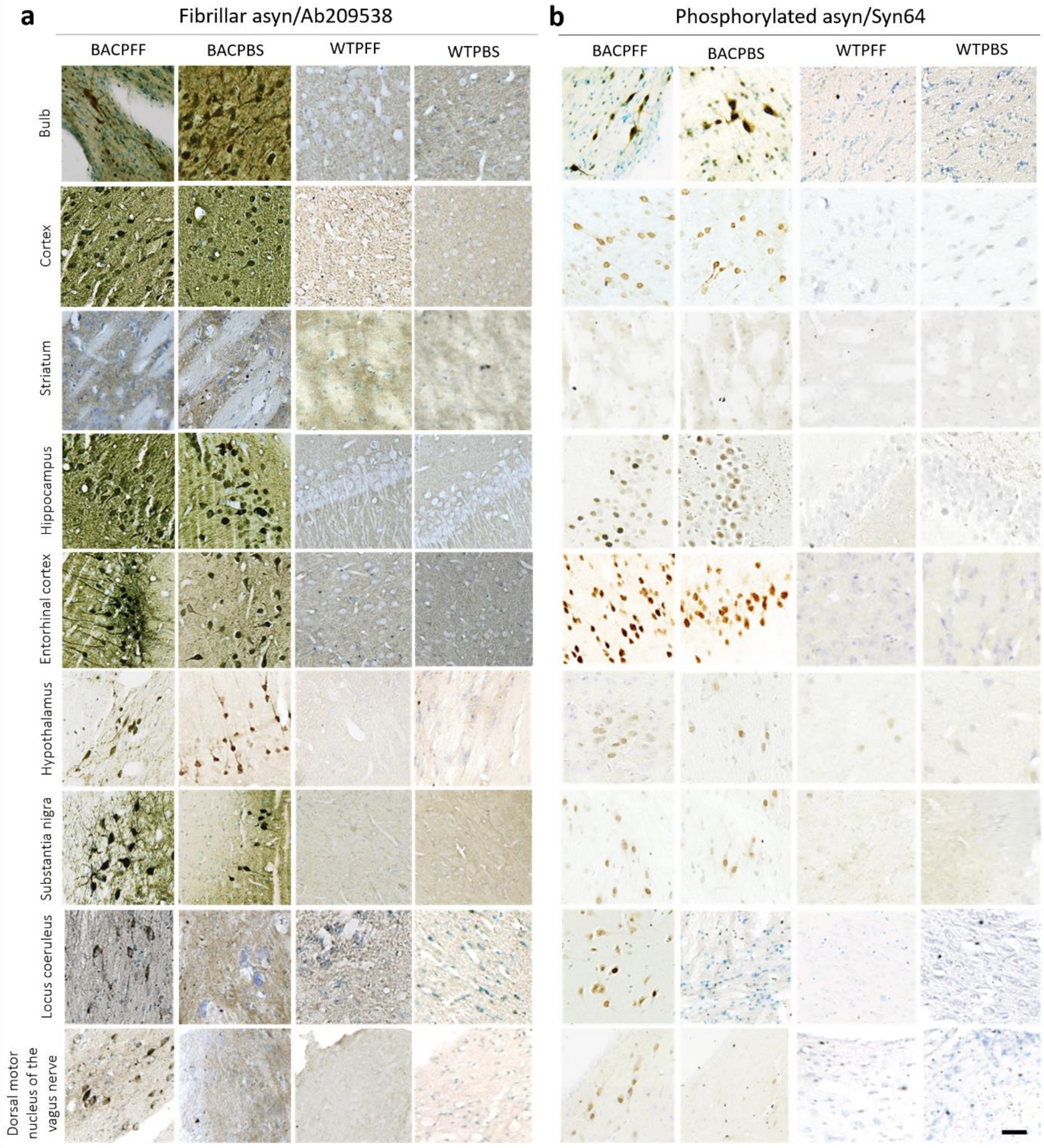

Online Resource Fig. 3 Representative high-magnification photomicrographs of staining for fibrillar asyn (**a**, Ab209538) and phosphorylated asyn (**b**, Syn64) pathology in several brain areas of S129A PFF- and PBS-injected BAC rats and WT controls. Note that asyn pathology in the locus coeruleus and dorsal motor nucleus was seen only in the BACPFF group. WT rats did not show convincing pathology anywhere in the brain. Scale bar is 50µm

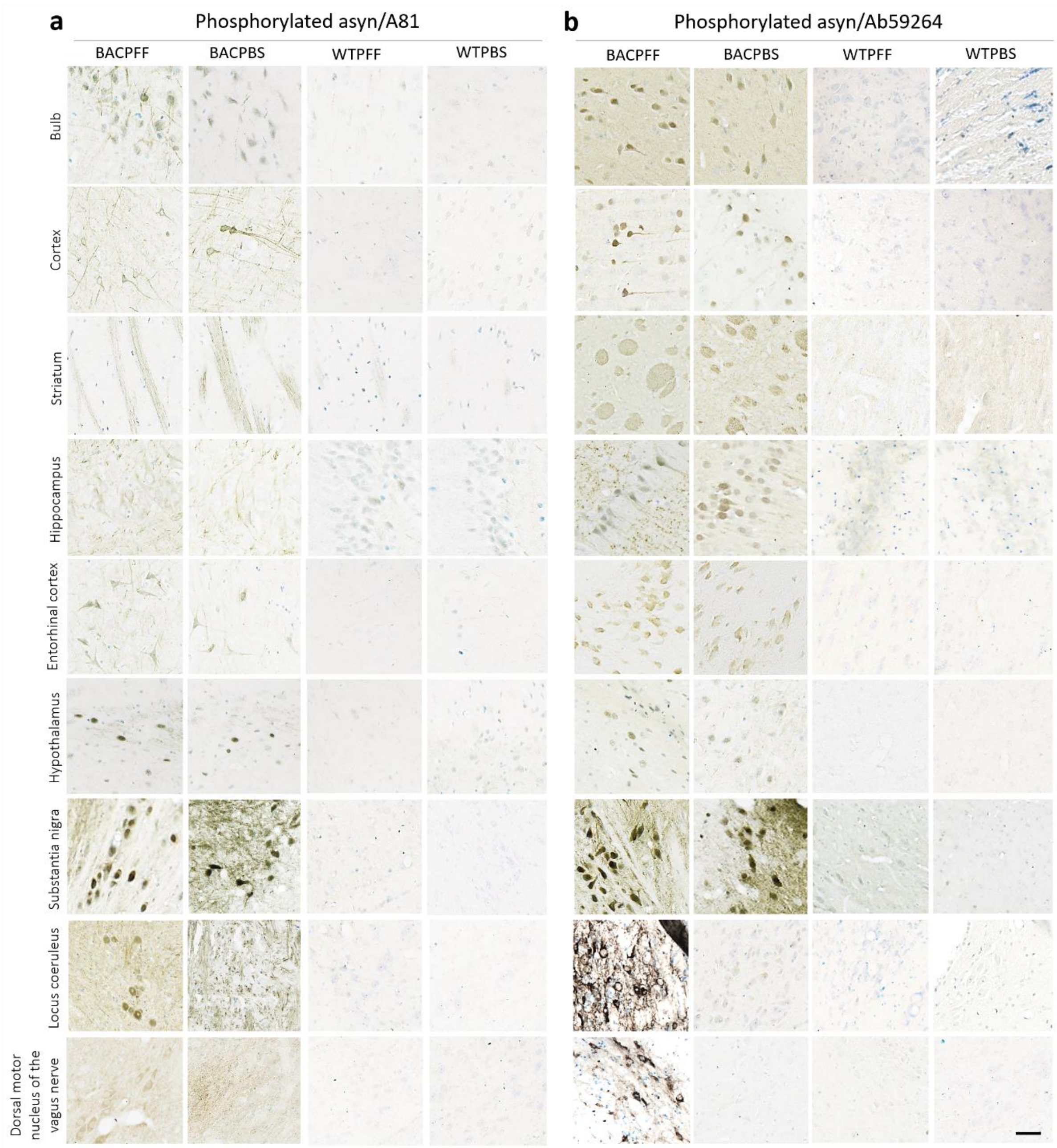

Online Resource Fig. 4 Representative high-magnification photomicrographs of staining for phosphorylated asyn (**a**, A81 and **b**, Ab59264) pathology in several brain areas of S129A PFF- and PBS-injected BAC rats and WT controls. Note that asyn pathology in the locus coeruleus and dorsal motor nucleus was seen only in the BACPFF group. WT rats did not show convincing pathology anywhere in the brain. Scale bar is 50µm

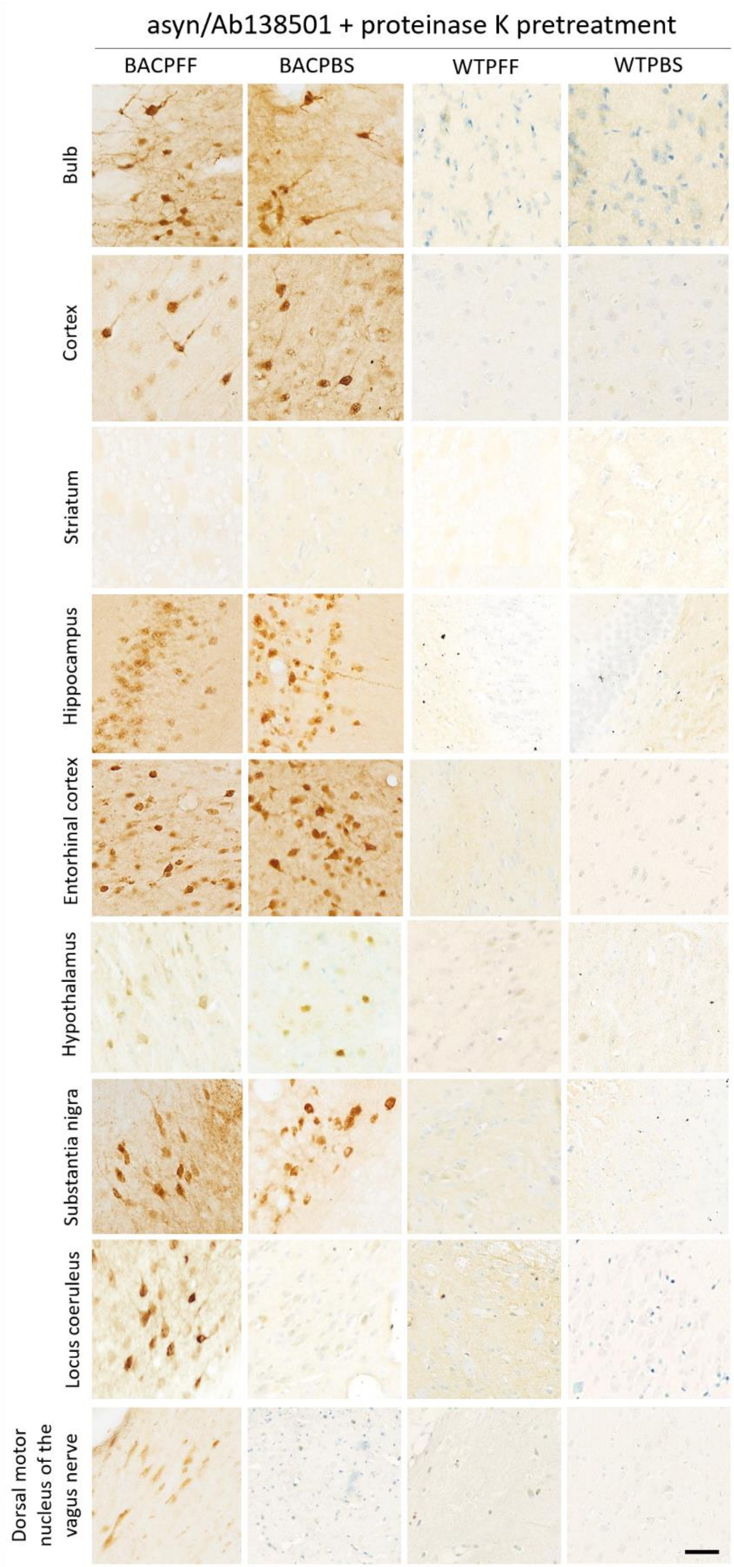

Online Resource Fig. 5 Representative high-magnification photomicrographs of staining for phosphorylated asyn (Ab138501 + Proteinase K pretreatment) pathology in several brain areas of S129A PFF- and PBS-injected BAC rats and WT controls. Note that asyn pathology in the locus coeruleus and dorsal motor nucleus was seen only in the BACPFF group. WT rats did not show convincing pathology anywhere in the brain. Scale bar is 50 µm

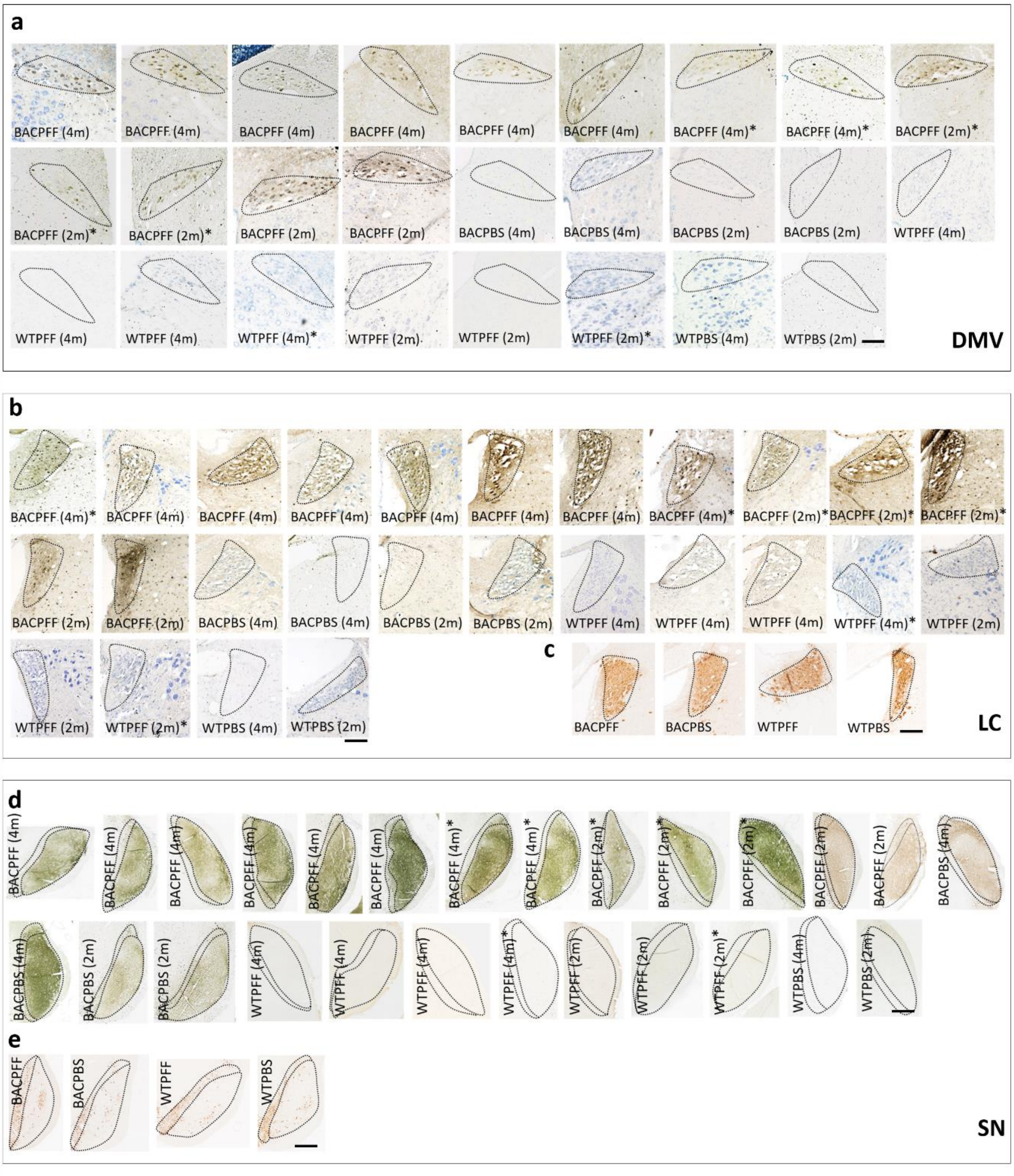

Online Resource Fig. 6 (**a**, **b**, **d**) Photomicrographs of staining for phosphorylated asyn (pSer129/Ab51253) pathology in the DMV (a), LC (b), and SN (d) of all S129A(*)- and WTPFF- and PBS-injected BAC rats and WT controls at two and four months (m) post injection. Note that asyn pathology in the DMV and LC was seen only in the BACPFF group. The amount of asyn pathology in the SN pars reticulata (SNr) of BACPFF rats was significantly higher than in the SNr of BACPBS rats. Similar pathology was seen in the SN pars compacta of BACPFF and BACPBS rats. WT rats did not show convincing pathology anywhere in the brain. Scale bar is 50µm. (**c**, **e**) Representative images of tyrosine hydroxylase distribution in the LC (c) and SN (e) of PFF- and PBS-injected BAC and WT rats. Scale bar DMV: 200 µm; scale bar LC: 150 µm; scale bar SN: 400 µm

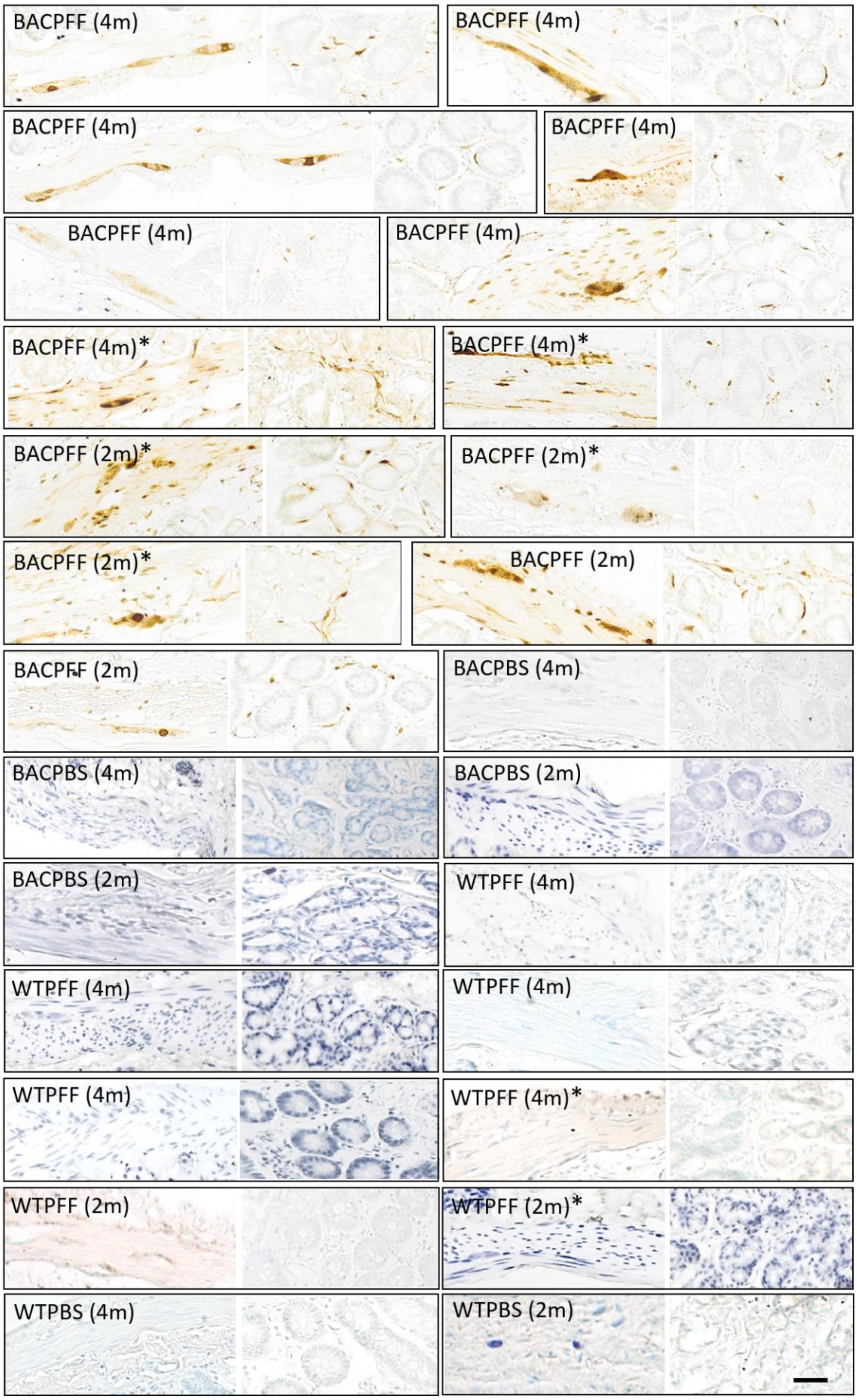

Online Resource Fig. 7 Distribution of asyn pathology (pSer129/Ab51253) in the duodenum of all S129A(*)- and WTPFF- and PBS-injected BAC rats and WT controls at two and four months (m) post injection. Representative high-magnification photomicrographs are shown of the ganglia and mucosa, left and right in each panel, respectively. Note that asyn pathology in the duodenum was only seen in the BACPFF group. Scale bar: 50 µm

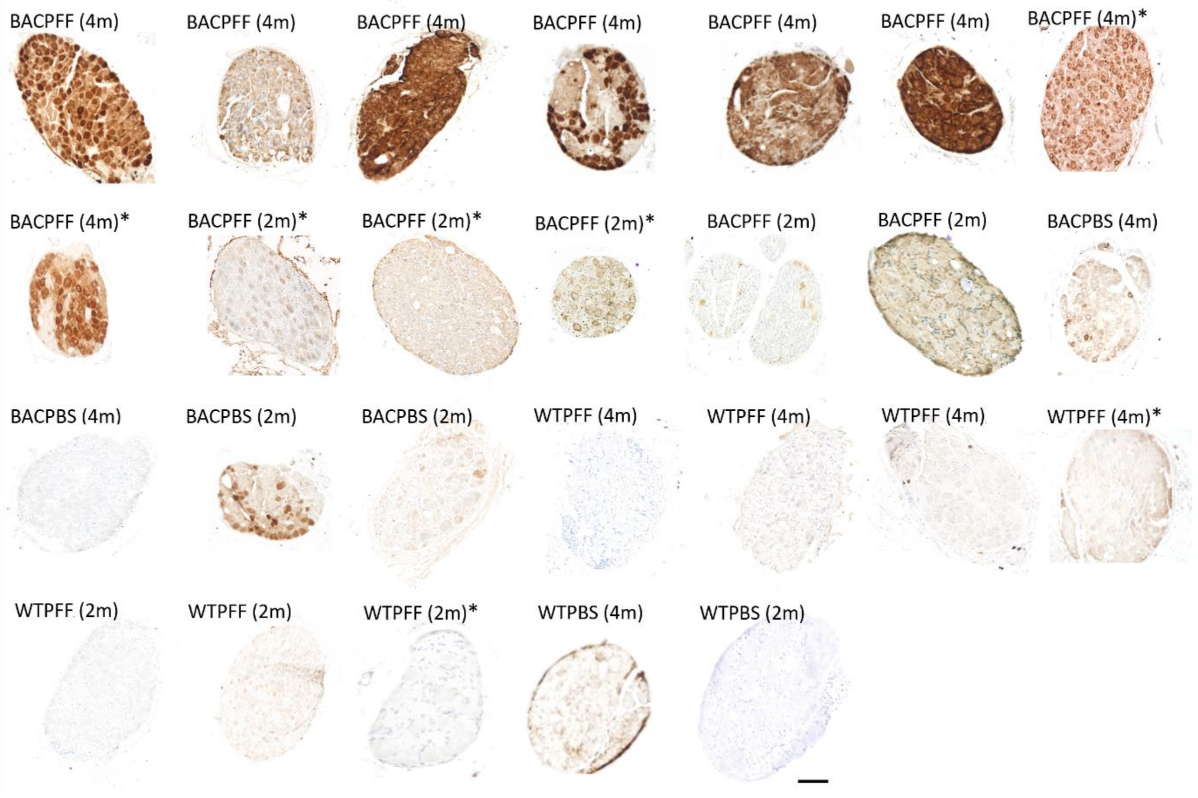

Online Resource Fig. 8 Distribution of asyn pathology (pSer129/Ab51253) in the celiac ganglia of all S129A(*)- and WTPFF- and PBS-injected BAC rats and WT controls at two and four months (m) post injection. The amount of asyn pathology in the BACPFF group was significantly higher than in the BACPBS group. No asyn pathology was seen in the WT rats. Scale bar: 200 µm

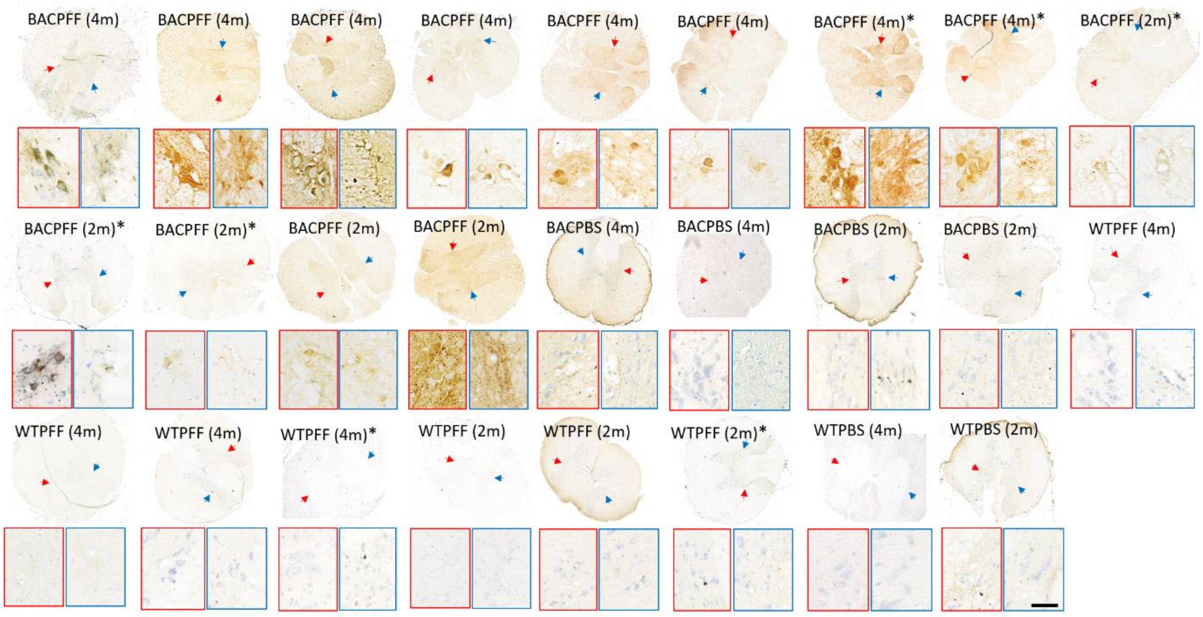

Online Resource Fig. 9 Distribution of asyn pathology (pSer129/Ab51253) in the IML of all S129A(*)- and WTPFF- and PBS-injected BAC rats and WT controls at two and four months (m) post injection. High-magnification photomicrographs of the left and right IML are shown in the bottom corners of each image. Note that BACPBS and WT rats did not show convincing pathology in the IML of the spinal cord. Scale bar: 50 µm

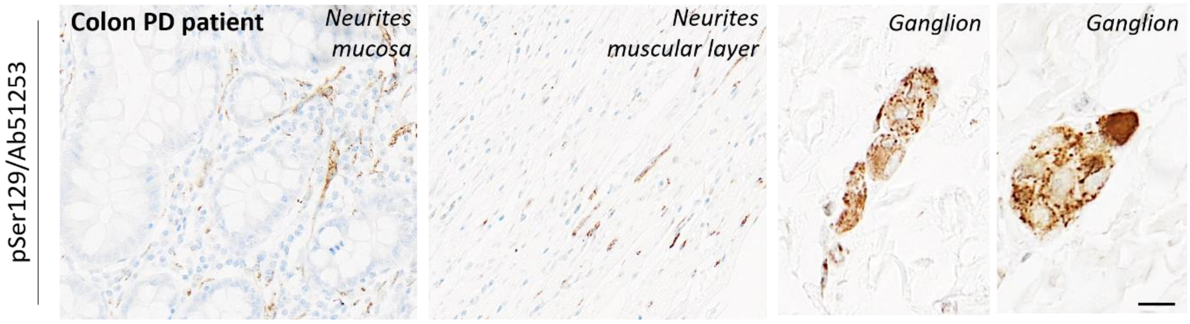

Online Resource Fig. 10 Distribution of asyn pathology (pSer129/Ab51253) in the colon of a PD patient. These results in human tissue are acquired with the same immunohistochemistry protocols as used for the animal tissue in this study. Representative high-magnification photomicrographs are shown of the ganglia and neurites. Scale bar: 50 µm

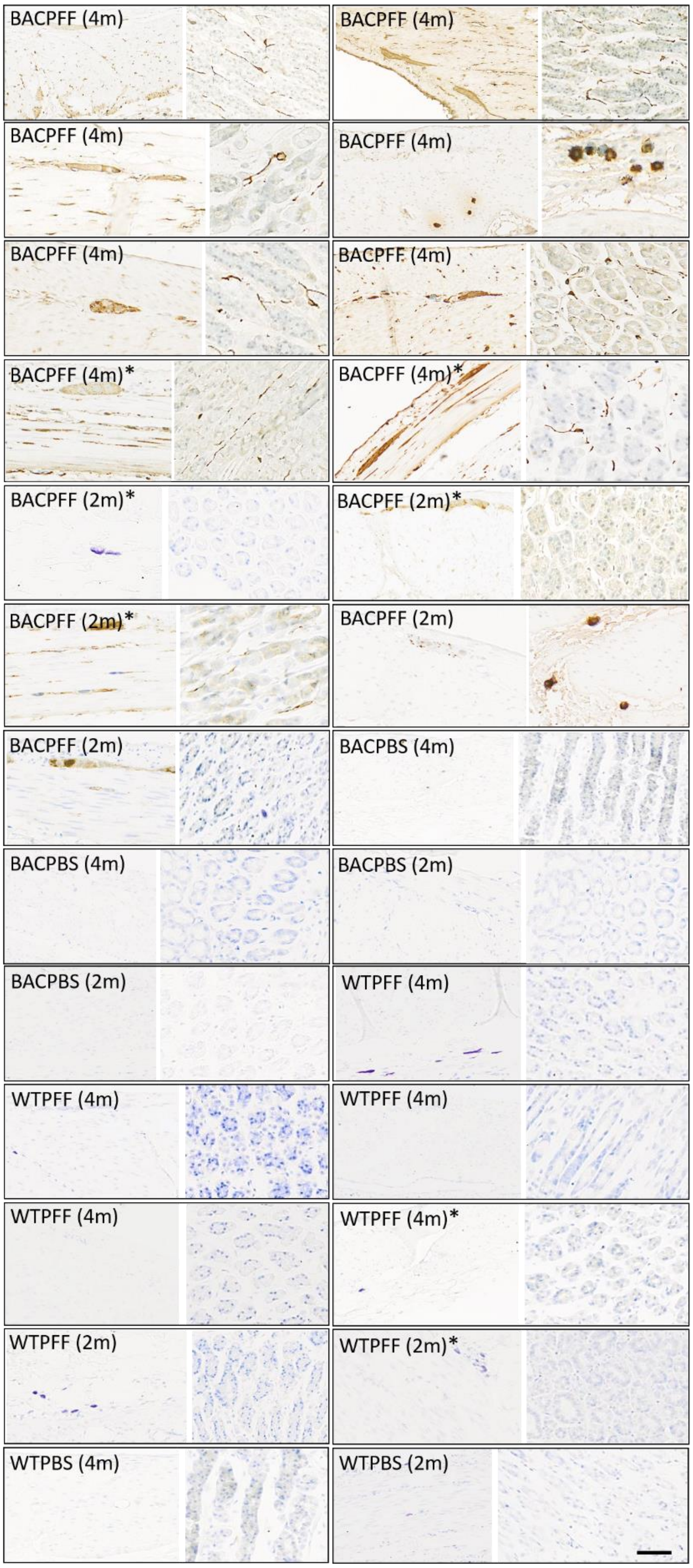

Online Resource Fig. 11 Distribution of asyn pathology (pSer129/Ab51253) in the stomach of all S129A(*)- and WTPFF- and PBS-injected BAC rats and WT controls at two and four months (m) post injection. Representative high-magnification photomicrographs are shown of the ganglia and mucosa (left and right in each panel, respectively). Note that asyn pathology in the stomach was only seen in the BACPFF group. Scale bar: 50 µm

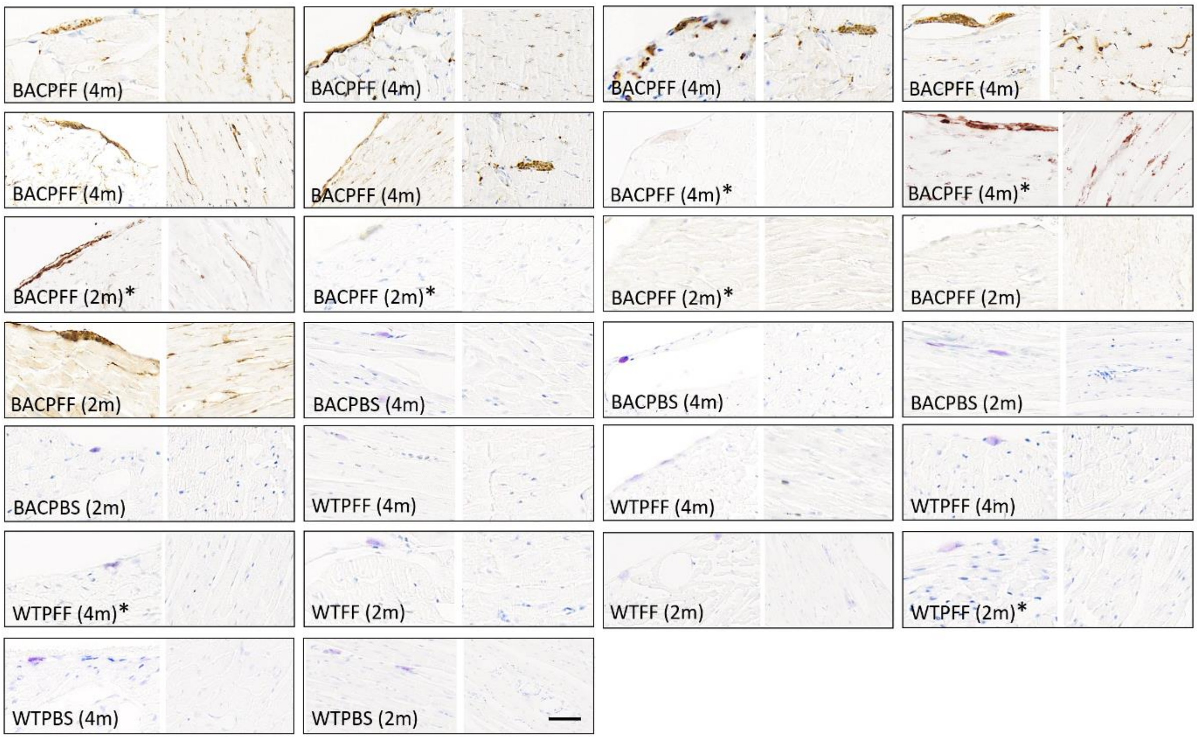

Online Resource Fig. 12 Distribution of asyn pathology (pSer129/Ab51253) in the heart of all S129A(*)- and WTPFF- and PBS-injected BAC rats and WT controls, at two and four months (m) post injection. Representative high-magnification photomicrographs are shown of the epicardium and myocardium (left and right in each panel, respectively). Note that asyn pathology in the heart was only seen in the BACPFF group. Scale bar: 50 µm
